# Differential Effects of Dietary White Meat and Red Meat on NAFLD Progression by Modulating Gut Microbiota and Metabolites in Rats

**DOI:** 10.1155/2022/6908934

**Published:** 2022-08-05

**Authors:** Juan Li, Yuting Li, Shufen Feng, Kaiyin He, Liliangzi Guo, Weiwei Chen, Min Wang, Lixian Zhong, Chutian Wu, Xiaojuan Peng, Shaohui Tang

**Affiliations:** ^1^Department of Gastroenterology, The First Affiliated Hospital, Jinan University, Guangzhou, Guangdong 510630, China; ^2^Department of Gastroenterology, The Third Affiliated Hospital, Jinzhou Medical University, Jinzhou, Liaoning 121001, China; ^3^Department of Endocrinology, Liuzhou People's Hospital, Liuzhou, Guangxi 545006, China

## Abstract

**Objective:**

To assess the effects of dietary white meat (grass carp and chicken) and red meat (pork and beef) on metabolic parameters, including the intestinal microbiota and its metabolites (SCFAs and bile acids) in NAFLD rats induced by high-fat diet.

**Methods:**

NAFLD rats were randomly assigned to five groups: NAFLD group, grass carp group, chicken group, pork group, and beef group (10 rats in each group), and these rats were fed for 8 weeks using the high-fat diet, grass carp-based diet, chicken-based diet, pork-based diet, and beef-based diet, respectively. At the end of the intervention, NAFLD-related metabolic indexes, intestinal flora, and its metabolites were measured.

**Results:**

The grass carp-based diet significantly improved hepatic pathological changes and glycolipid metabolism, and the chicken-based diet only partially improved the metabolic parameters. However, NAFLD progression was observed in the pork group and the beef group. What is more, the white meat-based diet-mediated changes in the enrichment of beneficial bacteria (such as *Lactobacillus* or *Akkermansia*), SCFAs, and unconjugated BAs (such as UDCA) and the depletion of pathogenic bacteria (such as *Bilophila* and *Prevotella_9*) and conjugated BAs were observed, while the red meat-based diet-induced changes in the enrichment of pathogenic bacteria (*Prevotella_9* or *Lachnospiraceae_UCG-010*) and conjugated BAs and the depletion of SCFAs and unconjugated BAs were found.

**Conclusion:**

The dietary white meat and red meat modulating gut microbiota and its metabolites may favor and aggravate NAFLD in rats, respectively.

## 1. Introduction

Nonalcoholic fatty liver disease (NAFLD) is the most common form of chronic liver disease worldwide, paralleling a worldwide increase in diabetes and metabolic syndrome [1]. NAFLD is a continuum of liver abnormalities from nonalcoholic fatty liver (NAFL), characterized by fat accumulation in the liver, to nonalcoholic steatohepatitis (NASH), associated with ballooning of hepatocytes, inflammation, and/or fibrosis. The presence of NASH increases the risks of liver and possibly non-liver-related adverse outcomes. The hepatic adverse outcomes may include cirrhosis, liver failure, and hepatocellular carcinoma, whereas non-liver-associated adverse outcomes are primarily related to increased cardiovascular disease and malignancy [2]. Although there has been steady progress in clarifying the pathogenesis of NAFLD, no agent is approved yet for this condition by the US Food and Drug Administration or the European Medicines Agency [3].

NAFLD treatment is currently warranted and driven by comprehensive lifestyle intervention including weight loss, reduction of calorie intake, changing dietary composition, and increment of exercise [4]. However, these treatment strategies for NAFLD face varying problems, such as poor compliance of patients, and a small percentage actually sticks with it for the long term. On the other hand, it may be a feasible and sustainable intervention for NAFLD patients to adjust their dietary structure and implement a healthy dietary pattern. It has been discovered that adherence to Mediterranean diets other than Western diets could significantly improve the fat content of the liver in NAFLD patients [5]. In addition, the change in some dietary components, such as increased fiber and omega-3 polyunsaturated fatty acid (*ω*-3 PUFA) intakes, effectively improves NAFLD and this effect is independent of caloric restriction and weight reduction [6, 7].

Meat including white meat (fish and chicken) and red meat (pork, beef, and mutton), which contains high-quality protein, is an important source of animal protein nutrient for Chinese people in the usual diet. Also, fish is rich in unsaturated fatty acids, especially n-3eicosapentaenoic acid and docosahexaenoic acid, and chicken meat has a lower amount of saturated fatty acids and a higher proportion of polyunsaturated fatty acids than red meat [8]. But red meat embodies significant amounts of saturated fatty acids and heme iron. Epidemiological studies have shown that fish intake can reduce the risk of NAFLD [9–11], whereas increased consumption of red meat and processed red meat is positively associated with the occurrence of NAFLD [12]. Moreover, chicken consumption showed an inconsistent effect on the occurrence of metabolic syndrome. The study by Noureddin et al. [13] showed that chicken meat seemed to increase the risk of NAFLD; on the contrary, the study by Gross et al. indicated that chicken consumption decreased the level of apolipoprotein B and total cholesterol and may be an alternative strategy for treating type 2 diabetes [8]. However, the effect of dietary white meat and red meat on NAFLD progression has not been studied.

There are more than 1000 species of bacteria in the human intestinal tract, and gut microbiota dysbiosis has vital influences on the occurrence of human diseases such as obesity, diabetes, and NAFLD [14, 15]. The changes in gut microbiota may disrupt the gut tight junctions, leading to increased gut permeability and LPS translocation. Increased LPS translocation induces “metabolic endotoxemia,” which triggers inflammatory reactions and insulin resistance and promotes the development of NAFLD [16]. A direct involvement of gut microbiota in the development of NAFLD is suggested by the finding that NAFLD can be delivered to germ-free mice by fecal microbiota transplantation (FMT) [17]. Gut microbiota-derived metabolites, such as short-chain fatty acids (SCFAs), and bile acids could regulate related receptors to reduce or exacerbate liver steatosis and inflammation [16]. Moreover, several studies have shown that dietary intake of saturated fatty acids and sugar is associated with the development of NAFLD, whereas dietary fiber and vitamin D might play a role in the improvement of NAFLD [10, 18] and the mechanism may involve the change of intestinal flora [19, 20]. However, it remains unclear whether white meat and red meat affect NAFLD progression by modulating gut microbiota and metabolites. The present study was performed to observe the effect of dietary white meat (grass carp and chicken) and red meat (pork and beef) on metabolic parameters, including the intestinal microbiota and their metabolites (SCFAs and bile acids) in NAFLD rats induced by high-fat diet.

## 2. Materials and Methods

See supplementary methods.

## 3. Results

### 3.1. Effects of Dietary White Meat and Red Meat on Metabolic Phenotypes in NAFLD Rats

**Figure 1:**
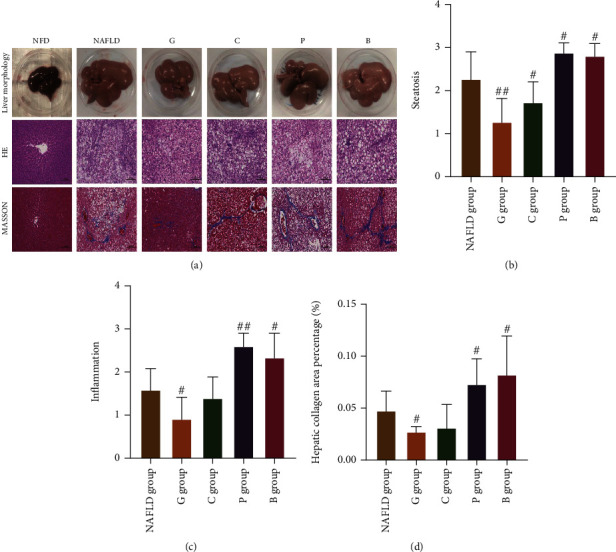
Hepatic pathological changes in laboratory rats. (a) Representative liver morphology and H&E-stained and MASSON-stained hepatic tissue sections (200x). (b) Histological scoring of steatosis. (c) Histological scoring of inflammation. (d) The quantitation of liver collagen fibers. The results are shown as mean ± SD. ∗*P* < 0.05 and ^∗∗^*P* < 0.01 vs the NFD group; ^#^*P* < 0.05 and ^##^*P* < 0.01 vs the NAFLD group. SD: standard deviation; NFD group: normal-fat diet group; NAFLD group: NAFLD control group; G group: grass carp group; C group: chicken group; P group: pork group; B group: beef group.

**Figure 2:**
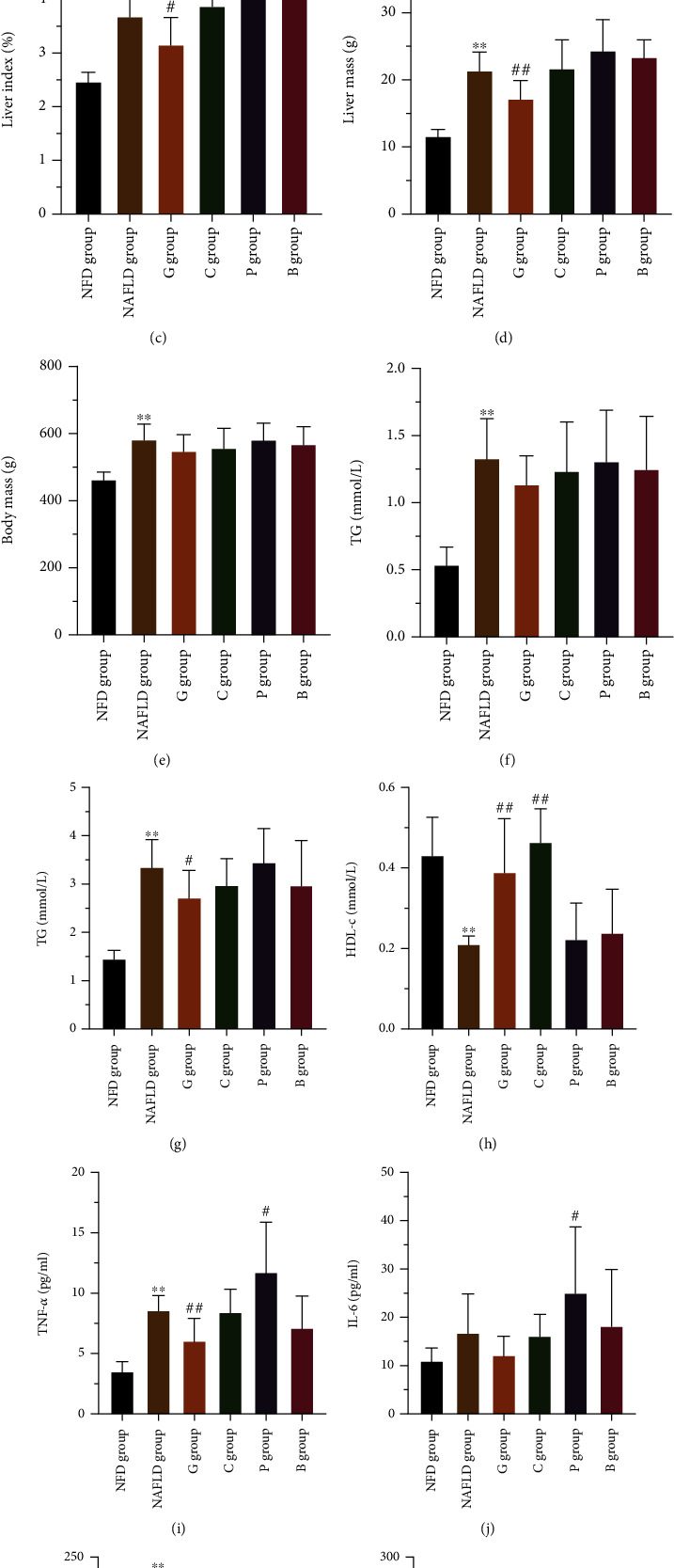
Dietary meat induced NAFLD phenotype changes in laboratory rats. The levels of (a) liver TG, (b) liver TC, (c) liver-to-body weight ratio (liver index), (d) liver mass, (e) body mass, (f) TG, (g) TC, (h) HDL-c, (i) TNF-*α*, (j) IL-6, (k) AST, and (l) ALP in rats fed with NFD, HFD, grass carp, chicken, pork, and beef were showed. The results are shown as mean ± SD. ^∗^*P* < 0.05 and ^∗∗^*P* < 0.01 vs the NFD group; ^#^*P* < 0.05 and ^##^*P* < 0.01 vs the NAFLD group. SD: standard deviation; NFD group: normal-fat diet group; NAFLD group: NAFLD control group; G group: grass carp group; C group: chicken group; P group: pork group; B group: beef group; AST: serum aspartate aminotransferase; ALP: serum alkaline phosphatase; TC: serum total cholesterol; TG: serum triglyceride; HDL-c: serum high-density lipoprotein cholesterol; TNF-*α*: serum tumor necrosis factor-*α*; IL-6: serum interleukin-6.

To examine the effects of dietary white meat and red meat on metabolic parameters of NAFLD, the rats with NAFLD were fed with white meat-based diets (grass carp-based and chicken-based diets), red meat-based diets (pork-based and beef-based diets), or a high-fat diet (Table 1). As showed in Figures 1(a) and 1(b), histological examination of liver sections showed that hepatic steatosis in rats fed with both the grass carp-based diet and the chicken-based diet as well as hepatic inflammation and fibrosis degree (Figures 1(c) and 1(d)) in rats fed with the grass carp-based diet was significantly improved, whereas hepatic steatosis, inflammation, and fibrosis degree in rats fed with both the pork-based diet and the beef-based diet were significantly exacerbated compared with those in rats fed with the HFD diet. Consistent with histological examination, reduced levels of hepatic triglycerides (TG) and total cholesterol (TC) in the grass carp group, a reduced hepatic TC level in the chicken group, and an elevated hepatic TG level in both the pork and the beef groups were observed compared with those in the NAFLD group (Figures 2(a) and 2(b)).

Along with the hepatic histopathologic changes, rats fed with the grass carp-based diet showed significantly decreased liver mass and liver index (liver-to-body mass ratio), while rats fed with both the pork-based diet and the beef-based diet showed significantly increased liver index compared with the NAFLD group (Figures 2(c) and 2(d)). There was no significant difference in body mass between the high-fat diet group and the four experimental diet groups (Figure 2(e)). In addition, serum metabolic parameters, including liver enzyme (alanine aminotransferase (ALT), aspartate transaminase (AST), glutamyl-transpeptidase (GGT), alkaline phosphatase (ALP)), blood lipids (high-density lipoprotein-cholesterol (HDL-c), low-density lipoprotein-cholesterol (LDL-c), TG, and TC), inflammatory factors (tumor necrosis factor *α* (TNF-*α*), C reactive protein (CRP), and interleukin-6 (IL-6)), and glycometabolic indicators (fasting blood glucose (FBG), fasting insulins (FINS), and insulin resistance (HOMA-IR)) were monitored at the end of this study. As shown in Figures 2(f)–2(l) and Figure S1A-G, the grass carp-based diet induced a remarkable improvement in AST, ALP, TC, HDL-C, TNF-*α*, FINS, and HOMA-IR and there were no significant differences in the levels of ALT, GGT, TG, LDL-c, IL-6, CRP, and FBG, compared with those in the NAFLD group; the rats fed with the chicken-based diet also showed a significant improvement in AST and HDL-C, and there were no significant differences in other phenotypes, compared with those in the NAFLD group. The levels of ALP, FBG, IL-6 and TNF-*α* in the pork-based diets group, and levels of ALP and FBG in the beef-based diets group were significantly increased compared with the NAFLD group, and there were no significant differences in other phenotypes. Collectively, these findings indicate that dietary white meat, especially grass carp, may drive the improvement of NAFLD phenotypes, whereas dietary red meat may exacerbate NAFLD.

### 3.2. Dietary White Meat and Red Meat Induce the Changes in Gut Microbiota in NAFLD Rats

**Figure 3:**
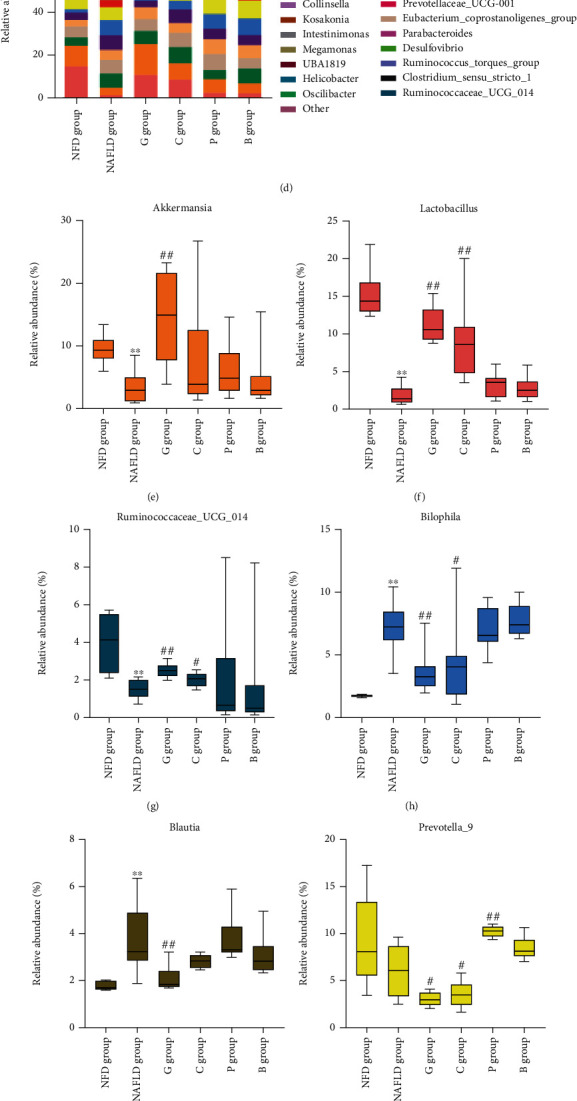
The structural changes of gut microbiota regulated by dietary intervention in laboratory rats. (a) Heatmap plot of the top 10 gut microbiota at the phylum level. The changes in the relative abundances of (b) *Verrucomicrobia* and (c) *Fusobacteria*. (d) Heatmap plot of the top 30 gut microbiota at the genus level in laboratory rats. Differences in the relative abundances of (e) *Akkermansia*, (f) *Lactobacillus*, (g) *Ruminococcaceae_UCG-014*, (h) *Bilophila*, (i) *Blautia*, (j) *Prevotella_9*, (k) *Ruminiclostridium_9*, and (l) *Lachnospiraceae_UCG-010* in the experimental diet groups. (m) Correlation heat map of bacterial microbiota with NAFLD phenotypes. The results are shown as median (interquartile range (IQR)) and compared by the Mann–Whitney *U* test. ^∗^*P* < 0.05 and ^∗∗^*P* < 0.01 vs the NFD group; ^#^*P* < 0.05 and ^##^*P* < 0.01 vs the NAFLD group. NFD group: normal-fat diet group; NAFLD group: NAFLD control group; G group: grass carp group; C group: chicken group; P group: pork group; B group: beef group.

In order to explore the potential role of the gut microbiota in mediating dietary white meat and red meat-induced improvement or aggravation in NAFLD phenotypes, we performed 16S rRNA gene sequencing in feces of NAFLD rats in five groups. At the phylum level (Figure 3(a)), the 10 most abundant microbiota in all groups were *Firmicutes*, *Bacteroidetes*, *Proteobacteria*, *Verrucomicrobia*, *Actinobacteria*, *Epsilonbacteraeota*, *Cyanobacteria*, *Fusobacteria*, *Patescibacteria*, and *Deferribacteres*. Increased *Verrucomicrobia* and *Fusobacteria* abundances were observed in the grass carp group compared with the NAFLD group (Figures 3(b) and 3(c)); but there were no significant differences in the abovementioned bacterial phyla between the chicken, pork, or beef groups and the NAFLD group (Figure S2).

At the genus level, top 30 dominant genera were presented in Figure 3(d) and the top 15 genera were analyzed. Compared with the NAFLD group, dietary grass carp intervention significantly increased the abundances of *Lactobacillus*, *Akkermansia*, *Phascolarctobacterium*, and *Ruminococcaceae_UCG-014* (Figures 3(e)–3(g) and Figure S3A) and decreased the abundances of *Bilophila*, *Prevotella_9*, *Blautia*, and *Alloprevotella* (Figures 3(h)–3(j) and Figure S3B) and dietary chicken intervention also significantly increased the abundances of *Lactobacillus* and *Ruminococcaceae_UCG-014* (Figures 3(f) and 3(g)) and decreased the abundances of *Bilophila* and *Prevotella_9* (Figures 3(h) and 3(j)) and there were no significant differences in other gut microbiotas, whereas dietary pork intervention increased the abundance of *Prevotella_9*, *Ruminiclostridium_9*, and *Lachnospiraceae_UCG-010* (Figures 3(j)–3(l)) and dietary beef intervention significantly increased the abundance of *Lachnospiraceae_UCG-010*, compared with that of the NAFLD group (*P* < 0.05) (Figure 3(l)), and there were no significant differences in other gut microbiotas.

Then, a correlation analysis was performed to determine the potential associations of bacterial abundance with NAFLD rat phenotypes (Figure 3(m)). We observed that *Lactobacillus*, *Akkermansia*, *Phascolarctobacterium*, and *Ruminococcaceae_UCG-014*, which were enriched in fecal samples of the rats fed with the grass carp-based diet or the chicken-based diet, were negatively correlated with several metabolic parameters (hepatic TC and TG, liver index, liver weight, AST, ALP, TNF-*α*, and the score of steatosis, inflammation, and fiber collagen area), while *Bilophila* and *Blautia*, which were depleted in the rats fed with the grass carp-based diet or the chicken-based diet, were positively correlated with the level of hepatic TC and TG, liver index, liver weight, body weight, ALT, AST, ALP, GGT, IL-6, TNF-*α*, FBG, HOMA-IR, and the score of steatosis, inflammation, and fiber collagen area. Moreover, *Prevotella_9* and *Lachnospiraceae_UCG-010*, which were enriched in fecal samples of the rats fed with the pork-based diet or the beef-based diet, were positively correlated with liver TG, HOMA-IR, and the score of steatosis, inflammation, and fiber collagen area.

Together, these results suggest that gut microbiota changes induced by dietary white meat and red meat may contribute to the alterations of metabolic phenotypes in NAFLD rats.

### 3.3. Dietary White Meat and Red Meat Affect Metabolic Phenotypes by Inducing Metabolite Alteration in NAFLD Rats

**Figure 4:**
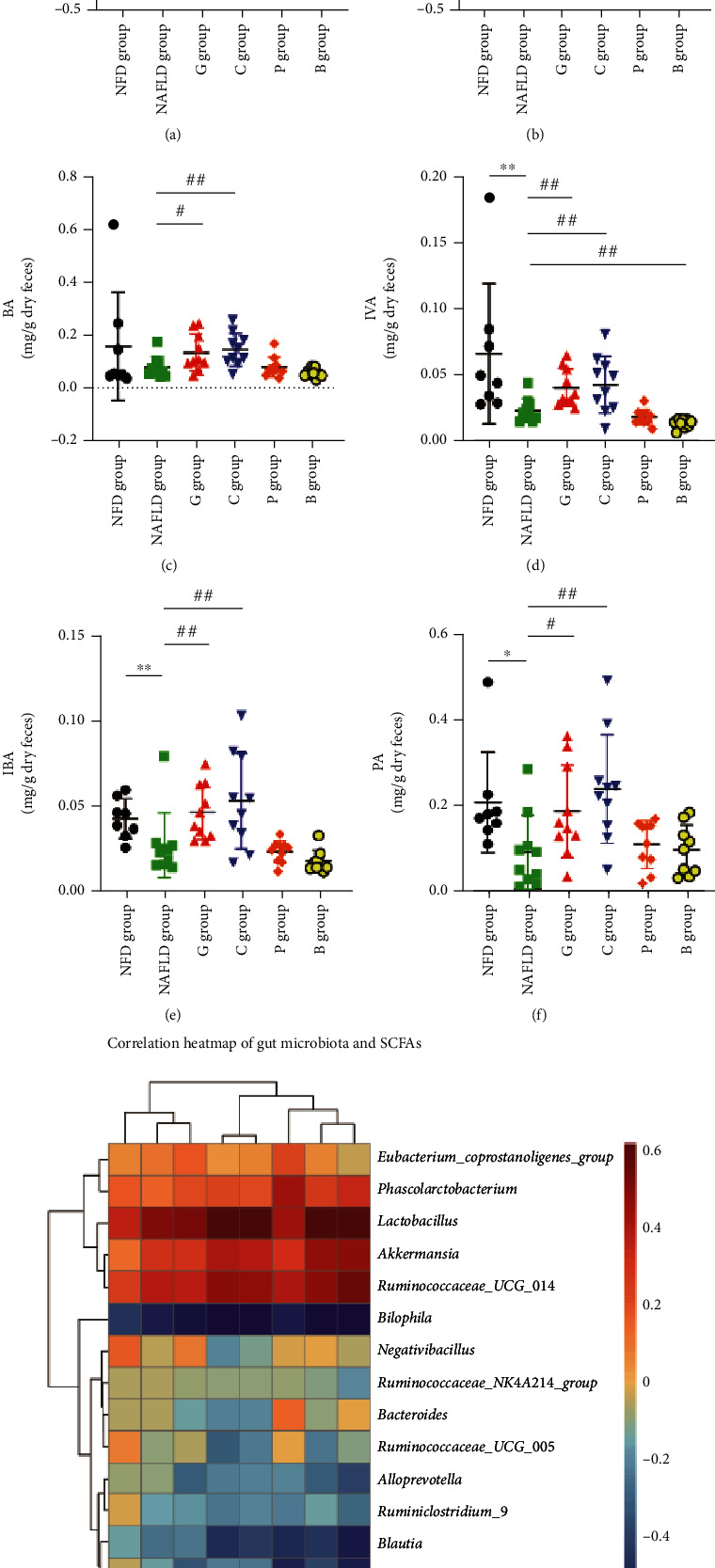
The changes of SCFAs regulated by dietary intervention in laboratory rats. The changes of (a) total SCFAs, (b) AA, (c) BA, (d) IVA, (e) IBA, and (f) PA in the experimental diet groups. (g) Correlation heat map of bacterial abundance and SCFAs. The results are shown as median (interquartile range (IQR)) and compared by the Mann–Whitney *U* test. ^∗^*P* < 0.05 and ^∗∗^*P* < 0.01 vs the NFD group; ^#^*P* < 0.05 and ^##^*P* < 0.01 vs the NAFLD group. NFD group: normal-fat diet group; NAFLD group: NAFLD control group; G group: grass carp group; C group: chicken group; P group: pork group; B group: beef group; SCFAs: short chain fat acids; AA: acetic acid; BA: butyric acid; IBA: isobutyric acid; IVA: isovaleric acid; PA: propionic acid.

To reveal metabolite alteration, related to the gut microbiome, which is potentially involved in metabolic phenotype alteration induced by dietary white meat and red meat, we performed metabolic profiling of feces from NAFLD rats. In this study, a total of 7 SCFA species were detected, including acetic acid (AA), propionic acid (PA), butyric acid (BA), isobutyric acid (IBA), valeric acid (VA), isovaleric acid (IVA), and hexanoic acid (HA). As shown in Figure 4 and Figure S4, compared with those in the NAFLD group, total SCFAs, AA, BA, PA, IBA, and IVA, except VA and HA in the grass carp group, and total SCFAs, AA, BA, PA, IBA, VA, and IVA, except HA in the chicken group, were increased significantly, while HA in the pork group and VA, IVA, and HA in the beef group were significantly decreased, and there were no significant differences in other SCFAs.

**Figure 5:**
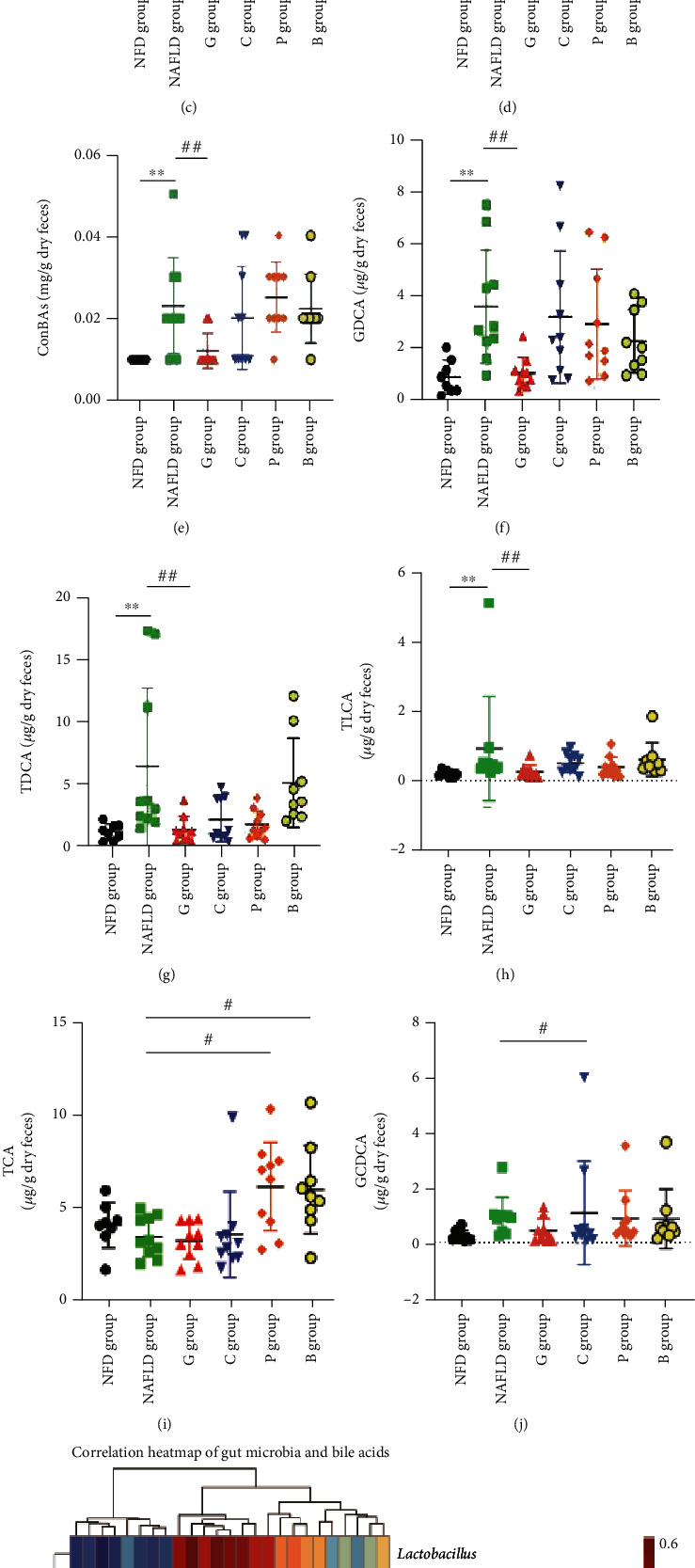
The changes of bile acids regulated by dietary intervention in laboratory rats. The changes of (a) total UnConBAs, (b) UDCA, (c) 7-KLCA, (d) 12-KLCA, (e) total ConBAs, (f) GCDCA, (g) TDCA, (h) TLCA, (i) TCA, and (j) GDCA in the experimental diet groups. (k) Correlation heat map of bacterial abundance and bile acids. The results are shown as median (interquartile range (IQR)) and compared by the Mann–Whitney *U* test. ^∗^*P* < 0.05 and ^∗∗^*P* < 0.01 vs the NFD group; ^#^*P* < 0.05 and ^##^*P* < 0.01 vs the NAFLD group; NFD group: normal-fat diet group; NAFLD group: NAFLD control group; G group: grass carp group; C group: chicken group; P group: pork group; B group: beef group; ConBAs: conjugated bile acids; UnConBAs: unconjugated bile acids; 12-KLCA: 12-ketolithocholic acid; GCDCA: glycochenodeoxycholic acid; GDCA: glycodeoxycholic acid; TCA: taurocholic acid; TDCA: taurodeoxycholic acid; UDCA: ursodeoxycholic acid; TLCA: taurolithocholic acid; 7-KLCA: 7-ketolithocholic acid.

There were 23 BA species to be detected in the present study, including 5 primary unconjugated BAs (cholic acid (CA), chenodeoxycholic acid (CDCA), *β*-muricholic acid (*β*-MCA), hyocholic acid (HCA), and *α*-muricholic acid (*α*-MCA)), 6 primary conjugated BAs (glycochenodeoxycholic acid (GCDCA), tauro-*β*-muricholic acid (T*β*-MCA), taurocholic acid (TCA), taurochenodeoxycholic acid (TCDCA), glycocholic acid (GCA), and glycohyocholic acid (GHCA)), 6 secondary unconjugated BAs (12-ketolithocholic acid (12-KLCA), 7-ketolithocholic acid (7-KLCA), lithocholic acid (LCA), deoxycholic acid (DCA), ursodeoxycholic acid (UDCA), and *ω*-muricholic acid (*ω*-MCA)), and 6 secondary conjugated BAs (glycodeoxycholic acid (GDCA), glycolithocholic acid (GLCA), glycoursodeoxycholic acid (GUDCA), taurodeoxycholic acid (TDCA), tauroursodeoxycholic acid (TUDCA), and taurolithocholic acid (TLCA)). As shown in Figure 5 and Figure S5, compared with those in the NAFLD group, total unconjugated bile acids (UnConBAs), UDCA, HCA, 7-KLCA, and 12-KLCA (Figures 5(a)–5(d) and Figure S5A) in the grass carp group and UDCA and 7-KLCA (Figures 5(b) and 5(c)) in the chicken group were upregulated significantly, whereas total conjugated bile acids (ConBAs), GDCA, GCA, TUDCA, TDCA, and TLCA (Figures 5(e)–5(h) and Figure S5B-C) in the grass carp group and GCDCA and TDCA (Figures 5(g) and 5(j)) in the chicken group were downregulated significantly, and there were no significant differences in other BAs. The conjugated bile acids TCA and T*β*-MCA (Figure 5(i) and Figure S5D) in the pork and beef groups were upregulated significantly, whereas unconjugated bile acid LCA (Figure S5E) was downregulated significantly in pork and beef groups, and there were no significant differences in other BAs.

Correlation analyses were performed to determine potential associations between microbe and metabolite changes (Figures 4(g) and 5(k)). Consistently, the results indicated that the grass carp group-enriched and the chicken group-enriched *Lactobacillus* was positively associated with total SCFAs, PA, BA, AA, IBA, VA, IVA, and HA, as well as total UnConBAs, UDCA, and 12-KLCA, and negatively correlated with total ConBAs, GDCA, GCA, TDCA, TUDCA, and TLCA. The grass carp group-enriched *Akkermansia* was positively associated with total SCFAs, AA, PA, IBA, IVA, and HA, as well as total UnConBAs and UDCA, and negatively correlated with GCA. The grass carp and chicken group-depleted *Bilophila* was negatively correlated with total SCFAs, PA, BA, AA, IBA, VA, IVA, and HA, as well as total UnConBAs, CA, UDCA, and 12-KLCA, whereas it was positively associated with total ConBAs, GCA, TDCA, GDCA, and GCDCA. The grass carp and chicken group-depleted and the pork group-enriched *Prevotella_9* was negatively correlated with VA, IBA, HA, CDCA, and 7-KLCA.

## 4. Discussion

Although dietary white meat and red meat have the different effects on the risk of NAFLD, information about the role and the underlined mechanisms of them in NAFLD progression is limited. In this study, we demonstrated for the first time that white meat-based diets, especially the grass carp-based diet, resulted in the significant improvements in hepatic steatosis and hepatic lipid profiles accompanied by the reduction in hepatic inflammation and/or fibrosis and the ameliorations of other metabolic parameters (liver index, liver enzyme, glycolipid metabolism indicators, or inflammatory factors), while red meat-based diets induced the aggravation of hepatic steatosis and hepatic lipid accumulation concomitant with the exacerbation of inflammation and/or fibrosis and several other metabolic phenotypes, independent of calorie intake and body weight. Taken together, these data suggest that dietary grass carp and chicken have the favorable effects on NAFLD rats; on the contrary, dietary pork and beef showed the unfavorable effects on NAFLD progression.

The different effects of dietary white meat and red meat on NAFLD rats in the study may be explained in part by the different nutrient contents in these diets. The report by Argo et al. showed that n-3 PUFA treatment led to the significant reduction in liver fat in NASH patients [21]; Capanni et al. revealed that supplementation with n-3 PUFAs improved biochemical, ultrasonographic, and hemodynamic features of liver steatosis of in patients with NAFLD [22]; Zibaeenezhad et al. found that n-3 PUFA supplements significantly ameliorated the blood lipid profile (TC, TG, and HDL-c) in patients with hyperlipidemia [23]. In addition, the diets enriched in monounsaturated fatty acids (MUFAs) enhanced lipid oxidation and reduced the liver fat content in patients with type 2 diabetes [24]; similarly, Errazuriz et al. reported that MUFA-rich diet decreased hepatic fat and improved both hepatic and total insulin sensitivity in people with prediabetes [25]. On the other hand, saturated fatty acids (SFA**)** have been shown to play an important role in the development and progression of NAFLD [26, 27]. Also, excess iron is potentially toxic to lead to oxidative stress and the increased serum ferritin level is closely related with insulin resistance, impaired glucose tolerance, and NAFLD pathogenesis [28]. Mayneris-Perxachs et al. [29] showed that serum ferritin levels were positively associated with liver fat accumulation. In an animal experiment, it was proved that iron deposits tended to increase with the degree of severity of liver fat changes [30]. The study by Atarashi et al. [31] also showed that dietary iron supplementation enhanced liver steatohepatitis induced by high-fat diet in rats. In the present study, the white meat-based diets had a higher amount of n-3 polyunsaturated fatty acids (n-3 PUFAs) and monounsaturated fatty acids (MUFA) than the HFD, and especially, the grass carp-based diet had the most amount of n-3 PUFAs (Table S5). The pork-based diet and the beef-based diet had significantly more amount of heme iron than that of the control group (Table 1). In addition, compared with the control diet, the pork-based diet had more amount of SFA and MUFA as well as a less amount of n-3 PUFA; the beef-based diet had more amount of MUFA and n-3 PUFA as well as less amount of SFA (Table S5). So, the improvement of the liver fat content and other phenotypes in the grass carp group and in the chicken-based group might be associated with significantly more n-3 PUFAs and MUFA in the experimental diets, whereas the progress of NAFLD phenotypes in the pork group might be associated with the higher levels of heme iron and SFA as well as the less level of n-3 PUFA, and that in the beef group might be associated with the significantly higher level of heme iron.

There is a growing body of evidence that gut microbiota plays an important role in the occurrence and development of diabetes, obesity, and NAFLD [32, 33]. Therefore, we explored the potential involvement of gut microbiota in mediating the different effects of dietary white meat and red meat on NAFLD rats. In this study, at the phylum level, the relative abundances of *Verrucomicrobia* and *Fusobacteria* significantly increased in the grass carp group compared with the NAFLD group. Consistent with our result, Pérez-Monter et al. indicated that Akkermansia that was the only member of *Verrucomicrobia* [34] significantly increased in the gut microbiota after inulin supplementation that improved diet-induced hepatic steatosis in mice [35]. However, Cortez-Pinto et al. showed that *Fusobacteria* markedly increased in a high-fat choline-deficient diet mice model of NASH, but less so with the addition of synbiotic that decreased liver fibrosis [36], which was contrary to our result, and further study is needed. In the level of genus, we observed that the relative abundances of *Lactobacillus* and *Akkermansia* were enriched, which are well known as probiotic bacterium to protect against obesity and obesity-linked metabolic syndrome [37], and the relative abundances of *Bilophila*, *Blautia*, and *Prevotella_9* were depleted, which were favored to grow by lipid dietary as the potentially pathogenic bacteria [36–39], in NAFLD rats fed with the grass carp-based diet or the chicken-based diet compared with the NAFLD group, along with the improvement in liver steatosis and other NAFLD-related metabolic phenotypes; conversely, we found that potentially pathogenic bacteria (such as *Prevotella_9* and *Lachnospiraceae_UCG-010*) were enriched in NAFLD rats fed with the pork-based diet or the beef-based diet compared with the NAFLD group, with the aggravation of hepatic steatosis and other metabolic parameters. Consistent with our results, Zhu et al. [38] indicated that the white meat (fish and chicken) group showed the higher relative abundance of *Lactobacillus* than the red meat group and nonmeat protein groups.

In addition to gut microbiota, different metabolites produced by commensal bacteria could be involved in the development of NAFLD [39, 40]. Gut microbiota-related metabolites, including SCFAs and BAs, reduce or exacerbate hepatic steatosis and inflammation by signaling from their homologous receptors. We therefore attempted to clarify the metabolites (SCFAs and BAs) that potentially mediate the different effects of dietary white meat and red meat on NAFLD progression.

In recent years, many studies have demonstrated the role of SCFAs produced by gut microbiota in the improvement of NAFLD, such as reducing proinflammatory cytokines, improving intestinal barrier function, and regulating immune function, insulin resistance, and glucose and lipid metabolism [41–43]. Metabolomic profiling in our study showed that total SCFAs, AA, BA, and PA were enriched in the grass carp group and in the chicken group. The report by Liang et al. [44] showed that compound probiotics significantly increased the levels of intestinal SCFAs in NAFLD rats and NAFLD phenotypes including liver TC and TG were significantly improved. An animal study suggested that SCFA administration in mice could suppress food intake and protect against a high-fat diet-induced weight gain and glucose intolerance [42]. The aforementioned findings support our study, in which the *Lactobacillus*, *Akkermansia*, *Phascolarctobacterium*, and *Ruminococcaceae_UCG-014*, which were enriched in fecal samples of rats fed with the grass carp-based diet or the chicken-based diet, have been reported to be positively associated with the content of SCFAs [33].

Bile acids are another important class of metabolites representing gut microbiota-host cometabolism. Bile acids could regulate the growth of gut bacteria, and gut bacteria metabolize bile acids to regulate host metabolism [39]. The gut-to-liver axis plays a critical role in the regulation. The results of BA profiling in our study indicated that the regulation of the BA signal might be involved in the effect of dietary white meat consumption on NAFLD. The levels of UnConBAs (such as UDCA and 7-KLCA) were enriched and ConBAs (such as GDCA and TDCA) were depleted in the grass carp group and in the chicken group, while UnConBAs (such as LCA) were depleted and ConBAs (such as TCA and T*β*-MCA) were enriched in the pork group and in the beef group compared with the NAFLD group. Consistent with our finding, the TCA, TCDCA, GCA, and GCDCA of ConBAs have been considered to cause hepatic lipid accumulation, hepatic apoptosis, and liver injury [39] and TCA, which enriched in the pork- and beef-based diets, has been considered to aggravate cholesterol-induced triglyceride accumulation [45]. The study by Puri et al. [46] suggested that the presence and severity of nonalcoholic steatohepatitis in patients are positively associated with the higher level of ConBAs, such as GCA, TCA, and TCDCA. A clinical trial revealed that type 2 diabetes patients treated with acarbose had an increased level of UnConBAs [47]. As consistent results as previously described, Zhang et al. [45] found that TCA, GCA, TUDCA, and TCDCA of ConBAs were enriched in high-fat high-cholesterol mice; the study by Lin et al. [48] indicated that HFD caused an increase in the levels of TDCA. The abovementioned findings were consistent with our results.

Collectively, our findings suggest that dietary grass carp and chicken intervention upregulates SCFAs and UnConBAs and downregulates ConBAs, thereby leading to the improvements in hepatic steatosis and other metabolic indicators; in contrast, dietary pork and beef intervention downregulates SCFAs and UnConBAs and upregulates ConBAs, in turn promoting NAFLD progression.

## 5. Conclusions

In conclusion, to the best of our knowledge, this study showed for the first time that demonstrated administration with the dietary grass carp, chicken, pork, and beef meat instead of casein and partial fat in high fat had the different effects on liver pathological changes and metabolic abnormalities in NAFLD rats. The dietary grass carp intervention could significantly improve hepatic NAFLD-related pathological changes and glycolipid metabolism, and the chicken-based diet may partially improve the metabolic parameters. However, NAFLD progression was observed in the pork group and the beef group. The mechanism responsible for the different effects may be at least partially correlated with the changes in gut microbiota composition and its metabolites, as indicated by discrepantly expanding the abundances of beneficial bacteria *Lactobacillus* or *Akkermansia* and inhibiting the growth of pathogenic bacteria *Bilophila* or *Blautia*. Then, the changes in gut microbiota caused the changes in gut metabolites, which were manifested as the increased levels of SCFAs and UnConBA and decreased ConBAs such as GCA, TDCA, GCDCA, and TLCA. Together, these findings suggest that dietary white meat modulating gut microbiota and its related metabolites may represent effective strategies in the improvement of phenotype in NAFLD rats.

## Figures and Tables

**Table 1:** Compositions of the experimental diets.

Group	Normal-fat diet	High-fat diet	Grass carp-based diet	Chicken-based diet	Pork-based diet	Beef-based diet
Composition (g/kg)						
Fish^a^			355.5			
Chicken^a^				375.2		
Pork^a^					382.2	
Beef^a^						313.8
Casein	180	257				
L-Cystein		3	3	3	3	3
Sucrose	265	100.0	87.2	84.8	83.5	93.1
Dyetrose	40	145.4	158.9	128.4	154.4	124.9
Cornstarch	240	0	0	0	0	0
Lard	14	310.3	228.4	243.7	210.6	296.6
Soybean oil	26	29.7	27.6	26.4	26.5	29.4
Cellulose	50	63	61.8	62.3	62.4	62.4
Mineral mix	62	57	55.6	55.2	56.1	55.3
Vitamin mix	10	13	13.3	12.9	12.7	13
Choline bitartrate	1.2	3	2.2	1.9	2.1	2.4
Analyzed (g/kg)^b^						
Crude protein	168	247.8	247.6	247.4	247.6	248.1
Fat	38	337.1	331.5	329.5	336.5	333.5
Ash content	41	22	71	75	76	75
Heme iron (mg)	0	0	3.2	3.8	10.4	19.1
Cholesterol	0	20	18.9	18.5	19.2	19.1
Total calories (kcal/kg)	3260	4606	4597	4621	4688	4685

^a^The amount of freeze-dried meat powder added into the experimental diets is based on the measurements of nitrogen in protein powder. Crude protein concentration was calculated using the formula *N*∗6.15 for casein and *N*∗5.6 for grass carp, chicken, pork, and beef. ^b^Analyzed values represent the mean of triplicate measurements.

## Data Availability

Data are in supplementary information files.

## Supplementary Materials

Supplementary Materials Supplementary Methods. Supplementary Figure. Figure S1: dietary meat induced NAFLD phenotype changes in laboratory rats. Figure S2: the structural changes of gut microbiota at the phylum level. Figure S3: the structural changes of gut microbiota at the genus level. Figure S4: the changes of SCFAs. Figure S5: the changes of bile acids. Supplementary Table. Table S1: compositions of the normal-fat diet and the high-fat diet. Table S2: amino acid compositions in dietary meat. Table S3: fatty acid compositions in dietary meat. Table S4: amino acid compositions in the experimental diets. Table S5: fatty acid compositions in the experimental diets.
