# Supplementary material for: Differential Effects of Dietary White Meat and Red Meat on NAFLD Progression by Modulating Gut Microbiota and Metabolites in Rats
Source: Oxid Med Cell Longev. 2022 Aug 5;2022:6908934. doi: 10.1155/2022/6908934 (PMC9410827; doi:10.1155/2022/6908934)
Supplement: Supplementary Materials — Supplementary Methods. Supplementary Figure. Figure S1: dietary meat induced NAFLD phenotype changes in laboratory rats. Figure S2: the structural changes of gut microbiota at the phylum level. Figure S3: the structural changes of gut microbiota at the genus level. Figure S4: the changes of SCFAs. Figure S5: the changes of bile acids. Supplementary Table. Table S1: compositions of the normal-fat diet and the high-fat diet. Table S2: amino acid compositions in dietary meat. Table S3: fatty acid compositions in dietary meat. Table S4: amino acid compositions in the experimental diets. Table S5: fatty acid compositions in the experimental diets. [file 6908934.f1.zip › supplementary figure.docx]

**Supplementary figures**


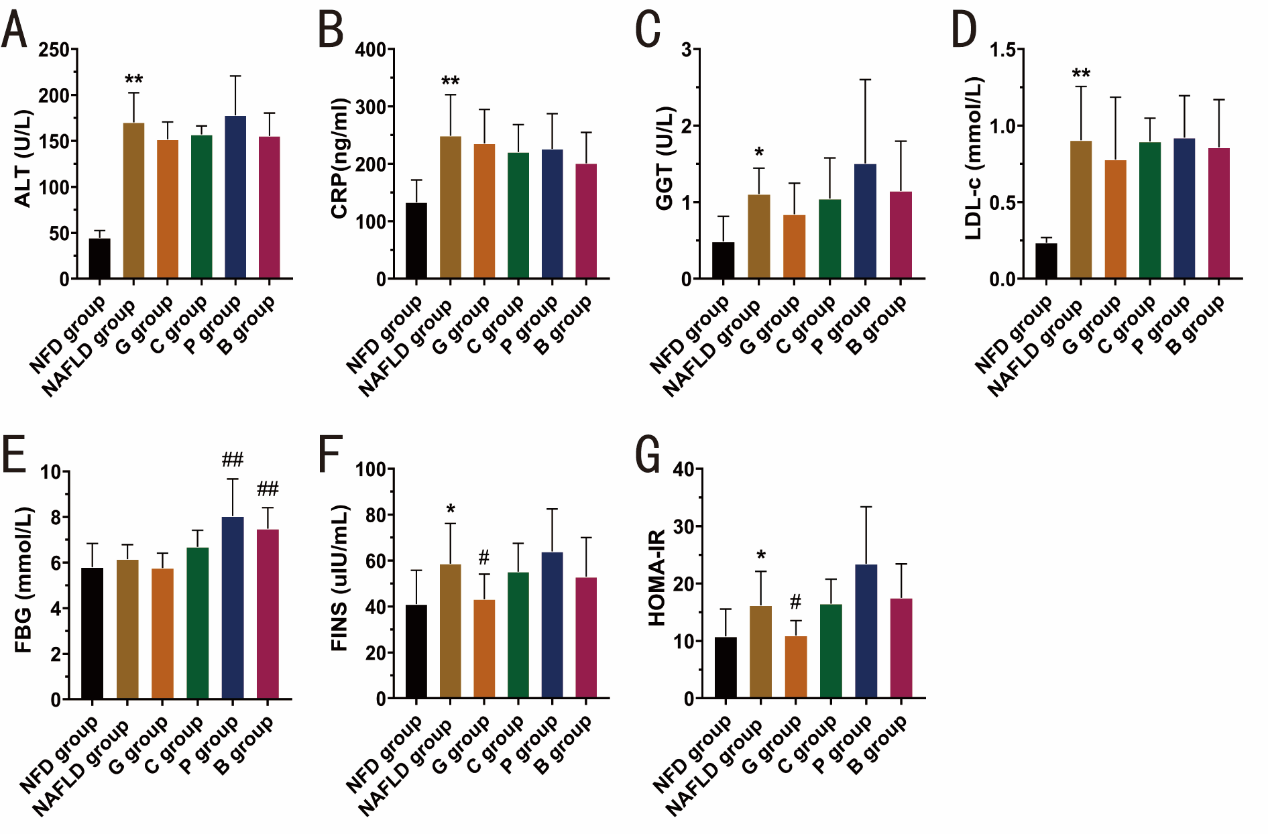


**Figure S1. Dietary meat induced NAFLD phenotype changes in laboratory rats.** The levels of (A) ALT, (B) CRP, (C) GGT, (D) LDL-c, (E) FBG, (F) FINS, (G) HOMA-IR in rats fed with NFD, HFD, grass carp, chicken, pork, and beef were showed. The results are shown as Mean±SD. *P <0.05, **P <0.01 vs NFD group; # P <0.05, ## P <0.01 vs NAFLD group. SD, standard deviation; NFD group, normal fat diet group; NAFLD group, NAFLD control group; G group, grass carp group; C group, chicken group; P group, pork group; B group, beef group; ALT, serum alanine aminotransferase; CRP, serum C-reactive protein; LDL-c, serum low-density lipoprotein cholesterol; FBG, fasting blood glucose; FINS, fasting insulins; HOMA-IR, insulin resistant index.


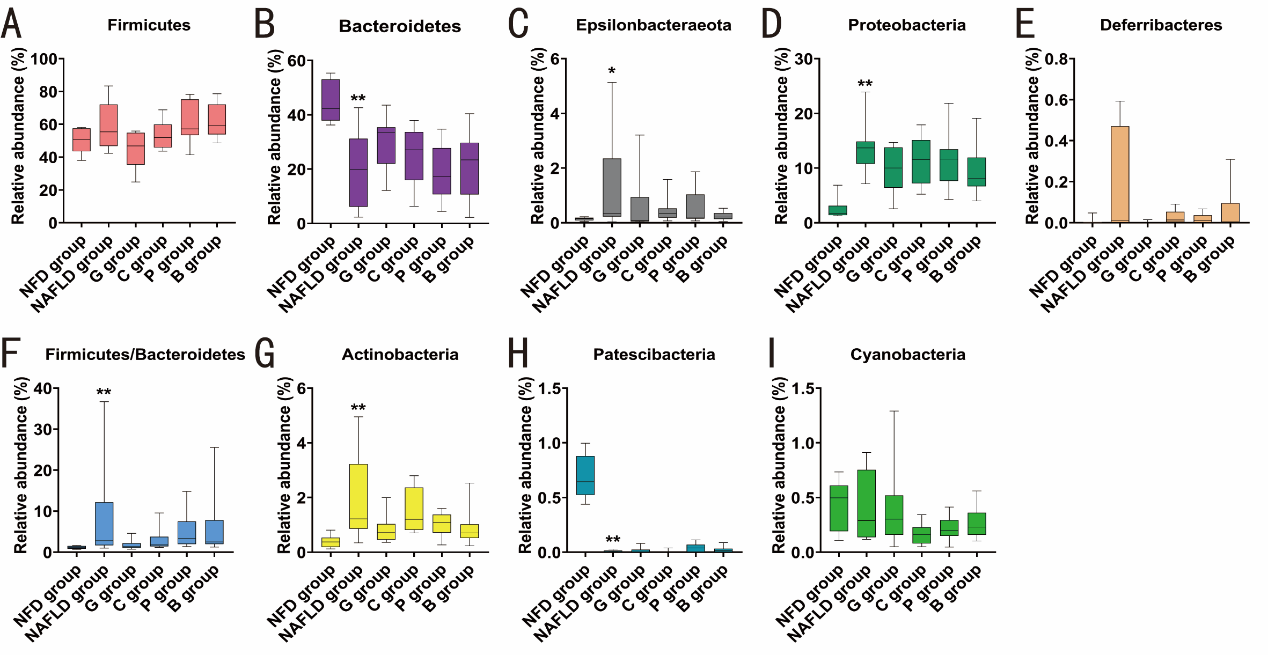


**Figure S2. The structural changes of gut microbiota regulated by dietary intervention at the phylum level in laboratory rats.** Differences in the relative abundances of (A) *Firmicutes,* (B) *Bacteroidetes,* (C) *Epsilonbacteraeota,* (D) *Proteobacteria,* (E) *Deferribacteres,* (F) the radio of *Firmicutes and Bacteroidetes,* (G) *Actinobacteria,* (H) *Patescibacteria* and (I) Cyanobacteria in the experimental diet groups. The results are shown as median (interquartile range, IQR) and compared by Mann-Whitney *U* test. *P <0.05, **P <0.01 vs NFD group; # P <0.05, ## P <0.01 vs NAFLD group. NFD group, normal fat diet group; NAFLD group, NAFLD control group; G group, grass carp group; C group, chicken group; P group, pork group; B group, beef group.


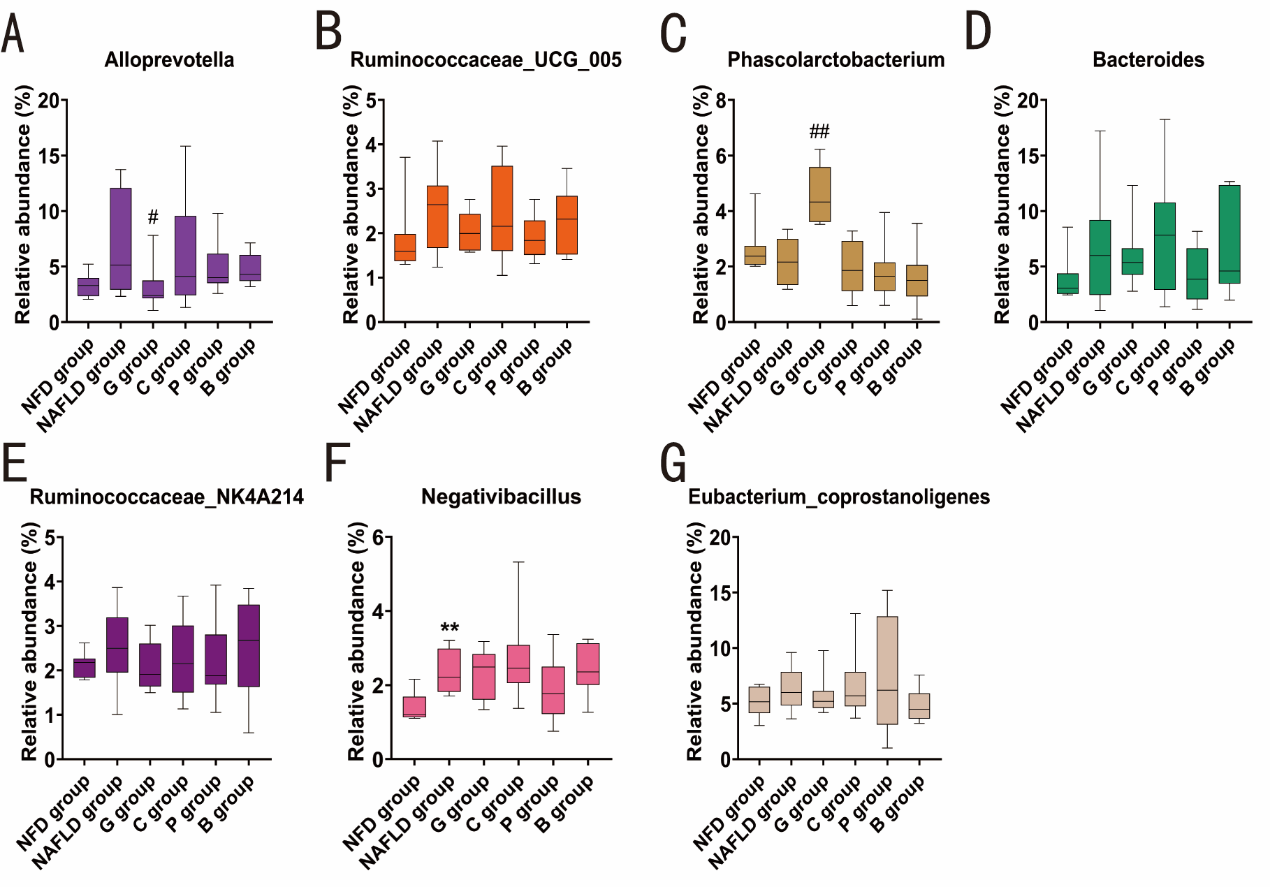


**Figure S3. The structural changes of gut microbiota regulated by dietary intervention at the genus level in laboratory rats.** Differences in the relative abundances of (A) *Alloprevotella,* (B) *Ruminococcaceae_UCG_005,* (C) *Phascolarctobacterium,* (D) *Bacteroides,* (E) *Ruminococcaceae_NK4A214,* (F) Negativibacillus*,* and (G) *Eubacterium_coprostanollgenes* in the experimental diet groups. The results are shown as median (interquartile range, IQR) and compared by Mann-Whitney *U* test. *P <0.05, **P <0.01 vs NFD group; # P <0.05, ## P <0.01 vs NAFLD group. NFD group, normal fat diet group; NAFLD group, NAFLD control group; G group, grass carp group; C group, chicken group; P group, pork group; B group, beef group.


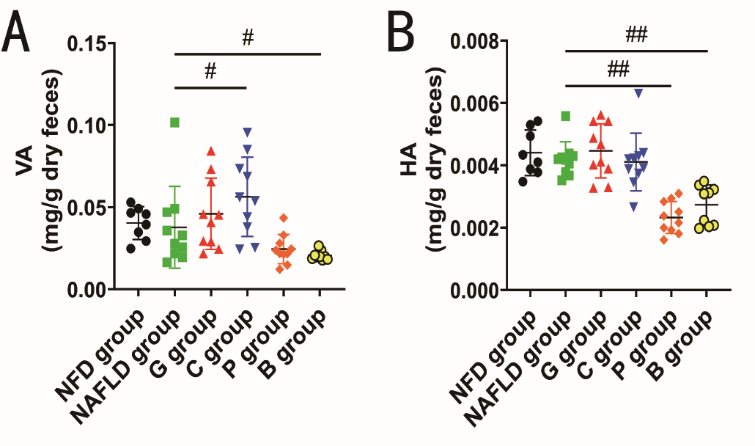


**Figure S4. The changes of SCFAs regulated by dietary intervention in laboratory rats.** The changes of (A) VA, and (B) HA in the experimental diet groups. The results are shown as median (interquartile range, IQR) and compared by Mann-Whitney *U* test. *P <0.05, **P <0.01 vs NFD group; # P <0.05, ## P <0.01 vs NAFLD group. NFD group, normal fat diet group; NAFLD group, NAFLD control group; G group, grass carp group; C group, chicken group; P group, pork group; B group, beef group; VA, [valeric](https://cn.bing.com/dict/search?q=Valeric&FORM=BDVSP6&mkt=zh-cn) [acid](https://cn.bing.com/dict/search?q=acid&FORM=BDVSP6&mkt=zh-cn); HA, Hexanoic acid.


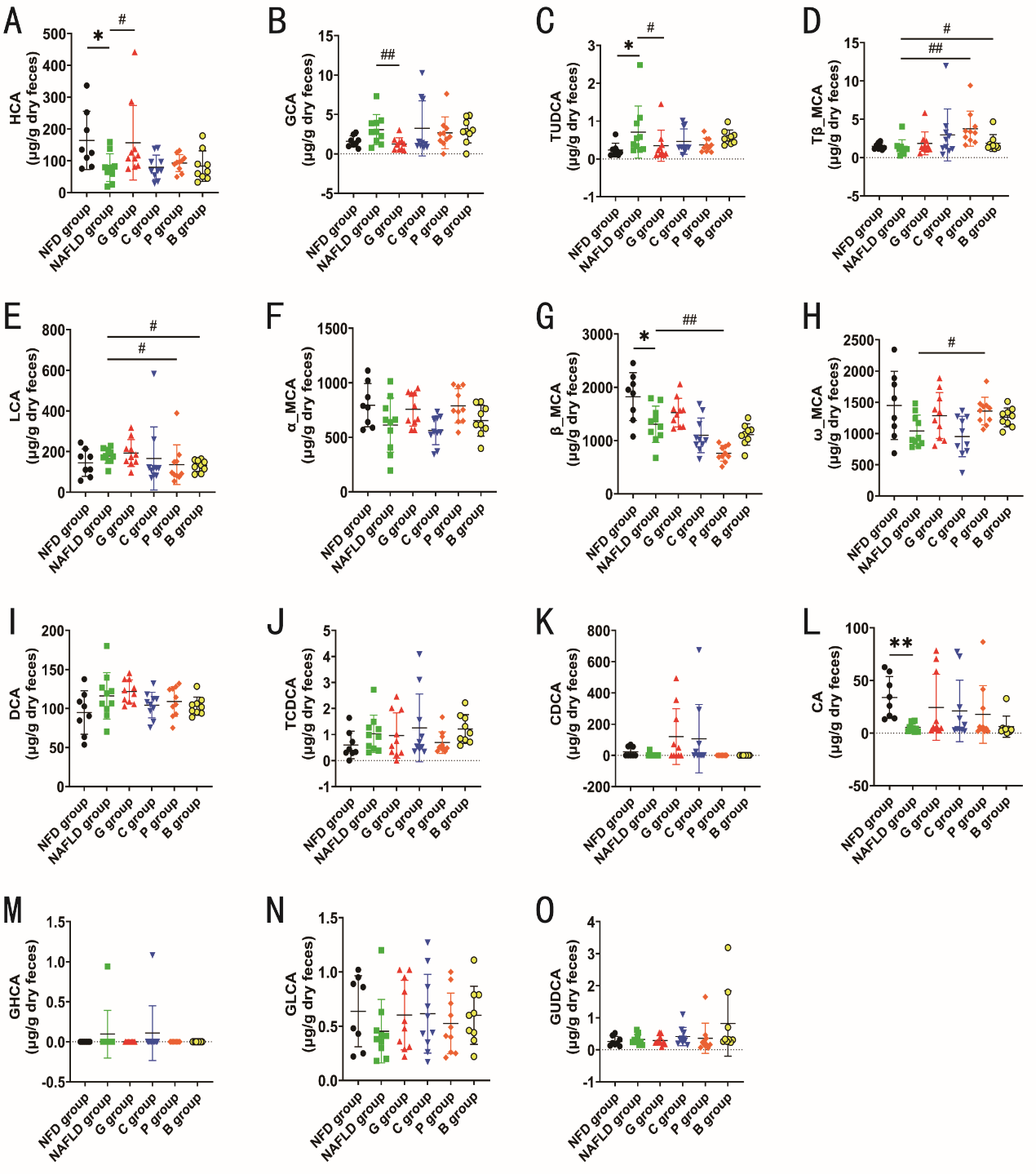


**Figure S5. The changes of bile acids regulated by dietary intervention in laboratory rats.** The changes of (A) HCA, (B) GCA, (C) TUDCA, (D) T-βMCA, (E) LCA, (F) α_MCA, (G) β_MCA, (H) ω-MCA, (I)DCA, (J) TCDCA, (K) CDCA, (L) CA, (M) GHCA, (N) GLCA, and (O)GUDCA in the experimental diet groups. The results are shown as median (interquartile range, IQR) and compared by Mann-Whitney *U* test. *P <0.05, **P <0.01 vs NFD group; # P <0.05, ## P <0.01 vs NAFLD group. NFD group, normal fat diet group; NAFLD group, NAFLD control group; G group, grass carp group; C group, chicken group; P group, pork group; B group, beef group; HCA, hyocholic acid; GCA, glycocholic acid; TUDCA, tauroursodeoxycholic acid; Tβ-MCA, tauro-β-muricholic acid; LCA, lithocholic acid; α-MCA, α-muricholic acid; β-MCA, β-muricholic acid; ω-MCA, ω-muricholic acid; DCA, deoxycholic acid; TCDCA, taurochenodeoxycholic acid; CDCA, chenodeoxycholic acid; CA, cholic acid; GHCA, glycohyocholic acid. GLCA, glycolithocholic acid; GUDCA, glycoursodeoxycholic acid.
