# Supplementary material for: Differential Effects of Dietary White Meat and Red Meat on NAFLD Progression by Modulating Gut Microbiota and Metabolites in Rats
Source: Oxid Med Cell Longev. 2022 Aug 5;2022:6908934. doi: 10.1155/2022/6908934 (PMC9410827; doi:10.1155/2022/6908934)
Supplement: Supplementary Materials — Supplementary Methods. Supplementary Figure. Figure S1: dietary meat induced NAFLD phenotype changes in laboratory rats. Figure S2: the structural changes of gut microbiota at the phylum level. Figure S3: the structural changes of gut microbiota at the genus level. Figure S4: the changes of SCFAs. Figure S5: the changes of bile acids. Supplementary Table. Table S1: compositions of the normal-fat diet and the high-fat diet. Table S2: amino acid compositions in dietary meat. Table S3: fatty acid compositions in dietary meat. Table S4: amino acid compositions in the experimental diets. Table S5: fatty acid compositions in the experimental diets. [file 6908934.f1.zip › supplementary methods-revised-2.docx]

**Supplementary Materials and Methods**

**Animal model**

A total of 62 male Sprague-Dawley (SD) rats (weight,180±20 g; age, 6 weeks) were purchased from the Southern Medical University (Guangzhou, China, quality certificate number: SCXK(Yue) 2016-0041). All the rats were housed under a temperature of 24±2℃, a relative humidity of 55±10%, and a 12 h light/dark cycle for 10 days before commencement of the animal experiment. All animal experiments were approved by the experimental animal ethics committee of Jinan University and were performed in accordance with the Guidelines for Care and Use of Laboratory Animals of Jinan University (NO. IACUC-20190517-08).

After 10 days acclimation, these rats were randomly divided into the control group with a normal-fat diet (NFD group, 4% fat, 54.5% carbohydrate, 18% protein, and 23.5% basic feed (w/w), n=11) and the model group with a high-fat diet (HFD group, 34% fat, 2% cholesterol, 26% carbohydrate, 26% protein, and 12% basic feed (w/w), n=51) to feed for 12 weeks (Table S1). Then, one rat was randomly selected from each of the two groups by using computer random numbers, and their livers were taken for pathological examination to confirm that NAFLD model was successfully established. The remaining 50 rats with NAFLD were randomly assigned to five groups: NAFLD control group, (NAFLD group, n=10), grass carp group (G group, n=10), chicken group (C group, n=10), pork group (P group, n=10), and beef group (B group, n=10).

**Experimental diets intervention**

Based on a high-fat background diet for rodents (Dyets, USA) (1), four different isonitrogenous and isoenergetic experimental diets were made containing protein from four different sources: grass carp fillets (grass carp-based diet), chicken breast fillets (chicken-based diet), pork sirloin fillets (pork-based diet), and beef sirloin fillets (beef-based diet). The fish, chicken, pork, and beef were obtained from the Shipai meat market (Guangzhou, China). Then, the four kinds of meat had been heated in a steamer to a core temperature of 70℃, freeze-dried and pulverized. In order to balance the macronutrient composition in the experimental diets, we measured amino acid compositions (Table S2) and fatty acid compositions (Table S3) in the freeze-dried meat as described previously(2). Apart from L-cystein supplementation (3g/kg), meat from the fish, chicken, pork, and beef was used as the sole protein source in the four experimental diets (Table 1). Particularly, both absolute and relative levels of n-3 PUFA were higher in fish meat and chicken meat, and therefore the n-6 to n-3 PUFA ratios were lower compared with the red meat (pork and beef) (Table S3). The amino acid compositions and fatty acid compositions in the experimental diets are shown in Table S4 and S5. Of note, the n-3 PUFA levels were higher in the grass carp-based diet and the chicken-based diet compared with the high fat diet (Table S5). The final diet recipes are shown in Table 1. These diets were prepared and stored at −20 °C. Before use, we measured total energy, fat content, crude protein, cholesterol and ash content of the diets according to the International Organization for Standardization ISO1443:1973, ISO 937:1978, ISO 5554:1978, ISO 11702:2016, and ISO 936:1998 (3-6), and we also measured heme iron as described previously(7). The result showed that heme iron in the grass carp-based diet and the chicken-based diet was significantly lower than that in the red meat-based diets (Table 1). The NAFLD rats in the NAFLD group, grass carp group, chicken group, pork group, and beef group were fed for 8 weeks using the high-fat diet, grass carp-based diet, chicken-based diet, pork-based diet, and beef-based diet, respectively. At the end of the intervention, NAFLD related metabolic indexes, intestinal flora and its metabolites were measured.

**Sample collection**

At the end of the 8-week experiment, overnight-fasted rats were anesthetized by 1% pentobarbital solution (30mg/kg), and the blood samples were collected from abdominal aortic. The blood samples were centrifuged for 15 min at 4 ◦C at 3000r, serum samples were then stored at -80 ℃ until detection. The liver tissues from each group of rats were rapidly removed, washed with physiological saline, weighed, and cut into three pieces; one piece was frozen in liquid nitrogen, one piece was fixed with 4% paraformaldehyde for histological examination, and the last piece was stored at -80 ℃ for later liver lipid detection. The liver index was calculated as follows: Liver index = (liver mass/body mass) ×100%.

During the experiment, 2 rats died in the NFD group caused by pentobarbital sodium.

**Serum and tissue biochemical measurements**

Serum was obtained by centrifuging whole blood samples. The fasting blood glucose (FBG), alanine aminotransferase (ALT), aspartate aminotransferase (AST), γ-glutamyl transpeptidase (GGT), alkaline phosphatase (ALP), total cholesterol (TC), triglyceride (TG), low-density lipoprotein cholesterol (LDL-c), high-density lipoprotein cholesterol (HDL-c) in serum, as well as the TG and TC in liver tissues were measured with commercial assay kits and the colorimetric enzymatic kits (Abcam, USA) according to the manufacturers’ instructions. The fasting insulins (FINS), C reactive protein (CRP), interleukin (IL)-6, and tumor necrosis factor (TNF)-α were determined by Mouse ELISA Kit (Abbkine Scientific, USA). The insulin resistant index (HOMA-IR) was also calculated by using the following formula: HOMA-IR=FBG (mmol/l) × FINS(μIU/ml)/22.5.

**Hepatic pathological examination**

The liver tissues were fixed overnight with 4% paraformaldehyde, then processed routinely, embedded in paraffin, sectioned to 5 μm thickness and stained with hematoxylin and eosin (H&E) or Masson trichrome (Masson) as previously described(8, 9). Then, liver histology was assessed by H&E staining and statistical evaluation was made as previously described(10). Steatosis was scored by low to medium power evaluation of parenchymal involvement according to the following criteria: 0 (<5%), 1 (5%-33%), 2 (33%-66%), or 3 (>66%). Inflammation was scored by overall assessment of all inflammatory foci according to the following criteria: 0 (No foci), 1 (<2 foci per 200X field), 2 (2-4 foci per 200X field), 3 (>4 foci per 200Xfield). Fibrosis was assessed by MASSON staining of paraffin embedded sections, and we used Image-Pro Plus 6.0 software (Media Cybernetics, Rockville, MD, USA) to quantitatively analyze hepatic collagen fibers (11). Two investigators who blinded to the treatment independently evaluated the slides and assigned scores for steatosis and inflammation.

**Gut microbiota analysis**

Fresh fecal samples of each rat were gathered, and frozen in liquid nitrogen immediately. The collected samples were stored at -80 ℃ until freeze-drying procedure and analyzed.

Freeze-drying procedure: Stool tubes were moved from the freezer to the drying chamber which connected to a freeze dryer. After drying, the fecal samples were moved from fecal tubes to a grinding chamber where they were ground. Then, the fine-grain dried fecal samples were preserved in cryotron at −80 °C until analysis.

To determine dietary white meat and red meat-induced gut microbiota alterations, DNA was extracted from fecal samples using HiPure Stool DNA Kit following manufacturer’s recommendations. The V3-V4 region of bacterial 16S rDNA gene was amplified using primers 341F (5’- CCT ACG GGN GGC WGC AG -3’) and 806R (5’-GGA CTA CHV GGG TAT CTA AT-3’). Sequencing libraries were generated using two-step PCR amplification method. The second round of amplification products was purified using AMPure XP Beads (Beckman Coulter, USA) and quantified using a QuantiFluorTM fluoromete (Promega, USA). At last, the library was sequenced on IlluminaHiSeq 2500 platform (Illumina, USA).

**SCFAs analysis**

Fecal SCFAs were measured using gas chromatography-mass spectrometer (GC-MS) (Agilment, USA). Freeze-dried feces (20mg) were diluted in 1mL [phosphoric](https://cn.bing.com/dict/search?q=phosphoric&FORM=BDVSP6&cc=cn) [acid](https://cn.bing.com/dict/search?q=acid&FORM=BDVSP6&cc=cn) solution (0.5% v/V). Then, the mixture was grilled by ball mill MM400 (Retsch, Germany) for 10s at 20Hz twice, vortexed for 10min (MIX-200, China), and treated by ultrasound for 5 min (incubated in ice water). The mixture was then centrifuged multiple times for 10 min at 4 °C at 12,000rmp/min, until the supernatant was clear. The supernatant fluid was centrifuged 100μL into the centrifuge tube, and added 500μL MTBE solvent with internal standard, vortex for 3 min, treated by ultrasound for 5 min (incubated in ice water). After centrifuged for 10 min at 4 °C at 12,000rmp/min, the supernatant was injected into an Agilent 7890B gas chromatography.

**Bile acid analysis**

Bile acids were measured using liquid chromatography-mass spectrometer (LC-MS) (SCIEX, Germany). Freeze-dried feces (20mg) were added in 200μL methanol homogenate. After vibrating at 2500 rpm for 10min, the mixture was transferred to a -20℃ refrigerator for 10min to precipitate protein. After centrifugation at 12000 r/min for 10 min, the supernatant was evaporated to dryness. Then, the extracts were reconstituted in 100 μL 50% methanol (V/V) and submitted to LC-MS analysis.

**Statistical analysis**

Statistical analysis was performed using SPSS 20.0 software (IBM Corp., USA). Differences between two groups were compared by independent-Samples *t* Test and Mann-Whitney *U* test. GraphPad Prism software (version 6.02) was used to plot graph, and R software was used to evaluate the correlation by Spearman correlation analysis. A difference with P<0.05 was considered significant.

**Reference**

1. Perry RJ, Resch JM, Douglass AM, Madara JC, Rabin-Court A, Kucukdereli H, et al. Leptin's Hunger-Suppressing Effects Are Mediated by the Hypothalamic-Pituitary-Adrenocortical Axis in Rodents. *Proceedings of the National Academy of Sciences of the United States of America* (2019) 116(27):13670-9. Epub 2019/06/20. doi: 10.1073/pnas.1901795116.

2. Tastesen HS, Keenan AH, Madsen L, Kristiansen K, Liaset B. Scallop Protein with Endogenous High Taurine and Glycine Content Prevents High-Fat, High-Sucrose-Induced Obesity and Improves Plasma Lipid Profile in Male C57bl/6j Mice. *Amino Acids* (2014) 46(7):1659-71. Epub 2014/03/25. doi: 10.1007/s00726-014-1715-1.

3. ISO 1443-1973. Determinación of Total Fat Content. International Standards Meat and Meat Products. International Organization for Standarization. Genève S.

4. ISO 937-1978. Determination of Protein Content. International Standards Meat and Meat Products.International Organization for Standarization. Genève S.

5. ISO 11702-2016. Determination of total sterols content. International Standards Animal and vegetable fats and oils. International Organization for Standarization. Genève S.

6. ISO 936-1998. Determination of ashes content. International Standards Meat and Meat Products. International Organization for Standarization. Genève S.

7. Zdanowska-Sasiadek Z, Marchewka J, Horbanczuk JO, Wierzbicka A, Lipinska P, Jozwik A, et al. Nutrients Composition in Fit Snacks Made from Ostrich, Beef and Chicken Dried Meat. *Molecules* (2018) 23(6). Epub 2018/05/26. doi: 10.3390/molecules23061267.

8. Larson K, Ho HH, Anumolu PL, Chen TM. Hematoxylin and Eosin Tissue Stain in Mohs Micrographic Surgery: A Review. *Dermatologic surgery : official publication for American Society for Dermatologic Surgery [et al]* (2011) 37(8):1089-99. Epub 2011/06/04. doi: 10.1111/j.1524-4725.2011.02051.x.

9. Chang JY, Kessler HP. Masson Trichrome Stain Helps Differentiate Myofibroma from Smooth Muscle Lesions in the Head and Neck Region. *Journal of the Formosan Medical Association = Taiwan yi zhi* (2008) 107(10):767-73. Epub 2008/10/18. doi: 10.1016/s0929-6646(08)60189-8.

10. Zhang X, Coker OO, Chu ES, Fu K, Lau HCH, Wang YX, et al. Dietary Cholesterol Drives Fatty Liver-Associated Liver Cancer by Modulating Gut Microbiota and Metabolites. *Gut* (2020). Epub 2020/07/23. doi: 10.1136/gutjnl-2019-319664.

11. Le Roy T, Llopis M, Lepage P, Bruneau A, Rabot S, Bevilacqua C, et al. Intestinal Microbiota Determines Development of Non-Alcoholic Fatty Liver Disease in Mice. *Gut* (2013) 62(12):1787-94. Epub 2012/12/01. doi: 10.1136/gutjnl-2012-303816.
