# Supplementary material for: Differential Effects of Dietary White Meat and Red Meat on NAFLD Progression by Modulating Gut Microbiota and Metabolites in Rats
Source: Oxid Med Cell Longev. 2022 Aug 5;2022:6908934. doi: 10.1155/2022/6908934 (PMC9410827; doi:10.1155/2022/6908934)
Supplement: Supplementary Materials — Supplementary Methods. Supplementary Figure. Figure S1: dietary meat induced NAFLD phenotype changes in laboratory rats. Figure S2: the structural changes of gut microbiota at the phylum level. Figure S3: the structural changes of gut microbiota at the genus level. Figure S4: the changes of SCFAs. Figure S5: the changes of bile acids. Supplementary Table. Table S1: compositions of the normal-fat diet and the high-fat diet. Table S2: amino acid compositions in dietary meat. Table S3: fatty acid compositions in dietary meat. Table S4: amino acid compositions in the experimental diets. Table S5: fatty acid compositions in the experimental diets. [file 6908934.f1.zip › supplementary table.docx]

**Supplementary Table**

**Table S1 Compositions of the normal fat diet and the high fat diet**

| **Components (Each 100g)** | **Normal Fat Diets (NFD)** | **High Fat Diets (HFD)** |
| --- | --- | --- |
| Energy(kcal) | 326 | 514 |
| Water(g) | 8 | 0 |
| Protein(g) | 18 | 26 |
| Fat(g) | 4 | 26 |
| Carbohydrat(g) | 54.5 | 24.5 |
| Cholesterol(g) | 0 | 1.9 |
| Ash(g) | 4 | 0 |
| Dietary fiber(g) | 5 | 6.3 |
| Vitamin A(g) | 0.1 | 1.3 |
| Calcium(g) | 1.5 | 2.34 |
| Mineral(g) | 4.8 | 3.4 |
| Choline(g) | 0.12 | 0.26 |

**Table S2 Amino acid compositions in dietary meat**

| **Amino acid** | **Freeze-dried grass carp** | | **Freeze-dried chicken** | | **Freeze-dried pork** | | **Freeze-dried beef** | |
| --- | --- | --- | --- | --- | --- | --- | --- | --- |
|  | **mg/g** | **%** | **mg/g** | **%** | **mg/g** | **%** | **mg/g** | **%** |
| **Essential** | 308.32 | 42.16 | 309.56 | 44.68 | 307.42 | 45.2 | 363.81 | 43.92 |
| Lysine (Lys) | 79.71 | 10.9 | 63.95 | 9.23 | 65.7 | 9.66 | 72.73 | 8.78 |
| Isoleucine (Ile) | 27.28 | 3.73 | 33.19 | 4.79 | 32.99 | 4.85 | 37.86 | 4.57 |
| Leucine (Leu) | 58.14 | 7.95 | 53.9 | 7.78 | 57.95 | 8.52 | 69.33 | 8.37 |
| Valine (Val) | 31.74 | 4.34 | 39.28 | 5.67 | 34.21 | 5.03 | 42.58 | 5.14 |
| Histidine (His) | 23.77 | 3.25 | 31.04 | 4.48 | 31.15 | 4.58 | 33.88 | 4.09 |
| Phenylalanine (Phe) | 29.98 | 4.1 | 28.96 | 4.18 | 34.21 | 5.03 | 42.58 | 5.14 |
| Methionine (Met) | 22.01 | 3.01 | 26.05 | 3.76 | 20.27 | 2.98 | 24.27 | 2.93 |
| Threonine (Thr) | 35.69 | 4.88 | 33.19 | 4.79 | 30.95 | 4.55 | 40.59 | 4.9 |
| **Non-Essential** | 422.9 | 57.83 | 383.28 | 55.32 | 372.71 | 54.8 | 464.54 | 56.08 |
| Asparagic (Asp) | 85.93 | 11.75 | 73.23 | 10.57 | 66.93 | 9.84 | 80.1 | 9.67 |
| Alanine (Ala) | 47.17 | 6.45 | 48.22 | 6.96 | 39.04 | 5.74 | 49.04 | 5.92 |
| Serine (Ser) | 32.61 | 4.46 | 29.31 | 4.23 | 25.51 | 3.75 | 34.71 | 4.19 |
| Glutamic(Glu) | 118.47 | 16.2 | 111.06 | 16.03 | 113.17 | 16.64 | 138.75 | 16.75 |
| Arginine(Arg) | 25.52 | 3.49 | 48.57 | 7.01 | 48.43 | 7.12 | 54.51 | 6.58 |
| Proline(Pro) | 56.38 | 7.71 | 30 | 4.33 | 25.03 | 3.68 | 38.68 | 4.67 |
| Tyrosine(Tyr) | 18.06 | 2.47 | 4.64 | 0.67 | 26.12 | 3.84 | 31.73 | 3.83 |
| Glycine(Gly) | 38.76 | 5.3 | 38.25 | 5.52 | 28.5 | 4.19 | 37.03 | 4.47 |
| **Total amino acid** | 731.22 | 100 | 692.84 | 100 | 680.13 | 100 | 828.36 | 100 |

All values represent mean of triplicate measurements.

**Table S3 Fatty acid compositions in dietary meat**

| **Fatty acid** | **Freeze-dried grass carp** | | **Freeze-dried chicken** | | **Freeze-dried pork** | | **Freeze-dried beef** | |
| --- | --- | --- | --- | --- | --- | --- | --- | --- |
|  | **mg/g** | **%** | **mg/g** | **%** | **mg/g** | **%** | **mg/g** | **%** |
| **SFA** | 65.13 | 28.43 | 61.3 | 34.33 | 129.19 | 48.57 | 11.46 | 34.13 |
| C14:0 | 2.34 | 1.02 | 1 | 0.56 | 5.88 | 2.21 | 0.64 | 1.92 |
| C15:0 | 0.44 | 0.19 | - | - | - | - | 0.23 | 0.69 |
| C16:0 | 40.77 | 17.80 | 43.07 | 24.12 | 77.9 | 29.3 | 6.05 | 18.03 |
| C17:0 | 1.86 | 0.81 | - | - | - | - | 0.79 | 2.36 |
| C18:0 | 18.96 | 8.28 | 16.82 | 9.42 | 44.04 | 16.56 | 3.59 | 10.68 |
| C20:0 | 0.76 | 0.33 | 0.41 | 0.23 | 1.33 | 0.5 | 0.15 | 0.45 |
| **MUFA** | 72.43 | 31.62 | 70.87 | 39.69 | 115.73 | 43.51 | 12.93 | 38.5 |
| C16:1 | 9.74 | 4.25 | 7.37 | 4.13 | 5.8 | 2.18 | 1.07 | 3.18 |
| C18:1 | 62.14 | 27.13 | 62.7 | 35.11 | 108.31 | 40.72 | 11.78 | 35.08 |
| C20:1 | 0.55 | 0.24 | 0.8 | 0.45 | 1.62 | 0.61 | 0.08 | 0.24 |
| **PUFA** | 91.52 | 39.95 | 46.39 | 25.98 | 21.07 | 7.92 | 9.19 | 27.37 |
| C18:2 | 41.83 | 18.26 | 35.45 | 19.85 | 19.36 | 7.28 | 6.05 | 18.01 |
| C18:3 | - | - | 1.82 | 1.02 | 0.98 | 0.37 | 0.33 | 0.97 |
| C20:4 | 26.66 | 11.64 | 7.98 | 4.47 | 0.51 | 0.19 | 2.41 | 7.19 |
| C20:5 | 7.61 | 3.32 | 1.14 | 0.64 | 0.13 | 0.05 | 0.29 | 0.85 |
| C22:6 | 15.42 | 6.73 | - | - | 0.08 | 0.03 | 0.12 | 0.35 |
| **n-3 PUFA** | 23.02 | 10.05 | 2.96 | 1.66 | 0.21 | 0.08 | 0.4 | 1.2 |
| **n-6 PUFA** | 68.49 | 29.9 | 43.43 | 24.32 | 19.87 | 7.47 | 8.46 | 25.2 |
| **n-6 PUFA/**  **n-3 PUFA** | 2.98 |  | 14.67 |  | 94.62 |  | 21.15 |  |
| **Total fatty acids** | 229.08 | 100 | 178.56 | 100 | 265.99 | 100 | 33.58 | 100 |

SFA, saturated fatty acids; MUFA, monounsaturated fatty acids; PUFA, polyunsaturated fatty acids. All values represent mean of triplicate measurements.

**Table S4 Amino acid compositions in the experimental diets**

| **Amino acid** | **High fat diet** | | **Grass carp-based diet** | | **Chicken-based diet** | | **Pork-based diet** | | **Beef-based diet** | |
| --- | --- | --- | --- | --- | --- | --- | --- | --- | --- | --- |
|  | **mg/g** | **%** | **mg/g** | **%** | **mg/g** | **%** | **mg/g** | **%** | **mg/g** | **%** |
| **Essential** | 111.52 | 43.67% | 111.00 | 42.14 | 117.63 | 44.65 | 116.82 | 45.17 | 112.78 | 43.89 |
| Lysine (Lys) | 17.34 | 6.79 | 28.70 | 10.89 | 24.30 | 9.22 | 24.97 | 9.65 | 22.55 | 8.77 |
| Isoleucine (Ile) | 15.08 | 5.90 | 9.82 | 3.73 | 12.61 | 4.79 | 12.54 | 4.85 | 11.74 | 4.57 |
| Leucine (Leu) | 26.32 | 10.31 | 20.93 | 7.95 | 20.48 | 7.77 | 22.02 | 8.51 | 21.49 | 8.36 |
| Valine (Val) | 15.34 | 6.01 | 11.43 | 4.34 | 14.93 | 5.67 | 13.00 | 5.03 | 13.20 | 5.14 |
| Histidine (His) | 6.24 | 2.44 | 8.56 | 3.25 | 11.80 | 4.48 | 11.84 | 4.58 | 10.50 | 4.09 |
| Phenylalanine (Phe) | 12.48 | 4.89 | 10.79 | 4.10 | 11.00 | 4.18 | 13.00 | 5.03 | 13.20 | 5.14 |
| Methionine (Met) | 5.72 | 2.24 | 7.92 | 3.01 | 9.90 | 3.76 | 7.70 | 2.98 | 7.52 | 2.93 |
| Threonine (Thr) | 13 | 5.09 | 12.85 | 4.88 | 12.61 | 4.79 | 11.76 | 4.55 | 12.58 | 4.90 |
| **Non-Essential** | 143.87 | 56.33 | 152.42 | 57.86 | 145.81 | 55.35 | 141.80 | 54.83 | 144.19 | 56.11 |
| Asparagic (Asp) | 17.5 | 6.85 | 30.93 | 11.74 | 27.83 | 10.56 | 25.43 | 9.83 | 24.83 | 9.66 |
| Alanine (Ala) | 9.42 | 3.69 | 16.98 | 6.45 | 18.32 | 6.96 | 14.84 | 5.74 | 15.20 | 5.92 |
| Serine (Ser) | 13.78 | 5.40 | 11.74 | 4.46 | 11.14 | 4.23 | 9.69 | 3.75 | 10.76 | 4.19 |
| Glutamic (Glu) | 53.22 | 20.84 | 42.65 | 16.19 | 42.20 | 16.02 | 43.00 | 16.63 | 43.01 | 16.74 |
| Arginine (Arg) | 9.87 | 3.86 | 9.19 | 3.49 | 18.46 | 7.01 | 18.40 | 7.12 | 16.90 | 6.58 |
| Proline (Pro) | 24.18 | 9.47 | 20.30 | 7.71 | 11.40 | 4.33 | 9.51 | 3.68 | 11.99 | 4.67 |
| Tyrosine (Tyr) | 10.44 | 4.09 | 6.67 | 2.53 | 1.93 | 0.73 | 10.09 | 3.90 | 10.02 | 3.90 |
| Glycine (Gly) | 5.46 | 2.14 | 13.95 | 5.30 | 14.54 | 5.52 | 10.83 | 4.19 | 11.48 | 4.47 |
| **Total amino acid** | 255.39 | 100 | 263.41 | 100.00 | 263.45 | 100.00 | 258.62 | 100.00 | 256.98 | 100.00 |

All values represent mean of triplicate measurements.

**Table S5 Fatty acid compositions in the experimental diets**

| **Fatty acid** | **High fat diet** | | **Grass carp-based diet** | | **Chicken-based diet** | | **Pork-based diet** | | **Beef-based diet** | |
| --- | --- | --- | --- | --- | --- | --- | --- | --- | --- | --- |
|  | **mg/g** | **%** | **mg/g** | **%** | **mg/g** | **%** | **mg/g** | **%** | **mg/g** | **%** |
| **SFA** | 148.65 | 41.13 | 154.42 | 32.19 | 156.16 | 35.22 | 211.65 | 42.50 | 126.43 | 35.84 |
| C14:0 | 3.88 | 1.07 | 5.20 | 1.08 | 4.05 | 0.91 | 8.51 | 1.71 | 4.35 | 1.23 |
| C15:0 | 0.00 | 0.00 | 0.44 | 0.09 | 0.00 | 0.00 | 0.00 | 0.00 | 0.23 | 0.07 |
| C16:0 | 77.40 | 21.41 | 98.36 | 20.50 | 104.23 | 23.51 | 131.14 | 26.33 | 80.14 | 22.72 |
| C17:0 | 0.00 | 0.00 | 1.86 | 0.39 | 0.00 | 0.00 | 0.00 | 0.00 | 0.79 | 0.22 |
| C18:0 | 67.38 | 18.64 | 47.80 | 9.96 | 47.48 | 10.71 | 70.67 | 14.19 | 40.77 | 11.56 |
| C20:0 | 0.00 | 0.00 | 0.76 | 0.16 | 0.41 | 0.09 | 1.33 | 0.27 | 0.15 | 0.04 |
| **MUFA** | 143.56 | 39.72 | 179.46 | 37.41 | 184.43 | 41.59 | 214.63 | 43.09 | 150.40 | 42.63 |
| C16:1 | 6.98 | 1.93 | 14.88 | 3.10 | 12.85 | 2.90 | 10.54 | 2.12 | 7.74 | 2.19 |
| C18:1 | 134.32 | 37.16 | 162.36 | 33.84 | 169.00 | 38.11 | 200.93 | 40.34 | 140.41 | 39.80 |
| C20:1 | 2.26 | 0.63 | 2.22 | 0.46 | 2.58 | 0.58 | 3.16 | 0.63 | 2.25 | 0.64 |
| **PUFA** | 69.21 | 19.15 | 145.86 | 30.40 | 102.88 | 23.19 | 71.76 | 14.41 | 75.96 | 21.53 |
| C18:2 | 62.34 | 17.2 5 | 90.73 | 18.91 | 85.24 | 19.22 | 64.97 | 13.05 | 66.17 | 18.76 |
| C18:3 | 4.76 | 1.32 | 3.89 | 0.81 | 5.29 | 1.19 | 4.63 | 0.93 | 5.15 | 1.46 |
| C20:4 | 0.00 | 0.00 | 26.66 | 5.56 | 7.98 | 1.80 | 0.51 | 0.10 | 2.41 | 0.68 |
| C20:5 | 2.11 | 0.58 | 9.16 | 1.91 | 4.39 | 0.99 | 1.56 | 0.31 | 2.11 | 0.60 |
| C22:6 | 0.00 | 0.00 | 15.42 | 3.21 | 0.00 | 0.00 | 0.08 | 0.02 | 0.12 | 0.03 |
| **n-3 PUFA** | 2.11 | 0.58 | 24.58 | 5.12 | 4.39 | 0.99 | 1.64 | 0.33 | 2.23 | 0.69 |
| **n-6 PUFA** | 62.34 | 17.25 | 117.39 | 24.47 | 93.22 | 21.25 | 65.48 | 13.15 | 68.58 | 19.44 |
| **n-6 PUFA/**  **n-3 PUFA** | 29.55 |  | 4.78 |  | 21.34 |  | 39.89 |  | 30.75 |  |
| **Total fatty acids** | 361.43 | 100.00 | 479.74 | 100.00 | 443.47 | 100.00 | 498.04 | 100.00 | 352.78 | 100.00 |

SFA, saturated fatty acids; MUFA, monounsaturated fatty acids; PUFA, polyunsaturated fatty acids. All values represent mean of triplicate measurements.
