# Supplementary material for: Differential Effects of Dietary White Meat and Red Meat on NAFLD Progression by Modulating Gut Microbiota and Metabolites in Rats
Source: Oxid Med Cell Longev. 2022 Aug 5;2022:6908934. doi: 10.1155/2022/6908934 (PMC9410827; doi:10.1155/2022/6908934)
Supplement: Supplementary Materials — Supplementary Methods. Supplementary Figure. Figure S1: dietary meat induced NAFLD phenotype changes in laboratory rats. Figure S2: the structural changes of gut microbiota at the phylum level. Figure S3: the structural changes of gut microbiota at the genus level. Figure S4: the changes of SCFAs. Figure S5: the changes of bile acids. Supplementary Table. Table S1: compositions of the normal-fat diet and the high-fat diet. Table S2: amino acid compositions in dietary meat. Table S3: fatty acid compositions in dietary meat. Table S4: amino acid compositions in the experimental diets. Table S5: fatty acid compositions in the experimental diets. [file 6908934.f1.zip › supplementary-original data.docx]

**NAFLD phenotype**

Index NFD-1 NFD-2 NFD-3 NFD-4 NFD-5 NFD-6 NFD-7 NFD-8 HFD-1 HFD-2 HFD-3 HFD-4 HFD-5 HFD-6 HFD-7 HFD-8 HFD-9 HFD-10 FS-1 FS-2 FS-3 FS-4 FS-5 FS-6 FS-7 FS-8 FS-9 FS-10 CK-1 CK-2 CK-3 CK-4 CK-5 CK-6 CK-7 CK-8 CK-9 CK-10 PG-1 PG-2 PG-3 PG-4 PG-5 PG-6 PG-7 PG-8 PG-9 PG-10 BF-1 BF-2 BF-3 BF-4 BF-5 BF-6 BF-7 BF-8 BF-9 BF-10

Liver Idex 2.505543 2.401848 2.681319 2.554113 2.607843 2.25 2.583519 2.378753 3.339192 4.008351 3.683333 4.302721 3.783784 3.230241 3.32737 3.404255 3.898917 3.854839 2.48954 3.680154 3.716475 3.785211 2.649007 3.636364 3.238866 2.85205 2.574751 2.794118 3.162393 3.398268 4.199656 4.651163 3.722772 3.921569 3.8 3.741259 4.433812 3.696319 3.492366 3.394495 4.093178 3.677298 4.788462 4.466192 4.117647 4.091681 4.740369 5.047319 4.424242 4.455067 3.81295 3.422939 3.976311 4.442413 3.711152 4.482759 4.460177 3.812317

ALT 45.214 35.669 41.594 41.356 54.941 48.522 54.338 33.315 141.302 153.875 186.317 174.907 166.432 171.909 131.249 241.503 142.567 192.63 126.659 163.55 127.899 150.927 153.618 135.835 180.246 141.876 177.148 160.009 160.121 166.969 162.179 168.577 151.328 157.679 147.011 149.867 165.451 141.749 125.129 231.087 141.254 148.214 149.289 223.019 168.236 235.089 143.348 213.574 145.594 160.582 170.364 151.25 141.591 216.701 147.46 132.494 129.348 158.828

AST 109.484 116.924 110.627 110.714 148.494 102.41 139.299 130.167 224.21 193.92 195.318 213.656 207.367 213.461 187.165 162.319 265.968 211.381 212.359 138.048 144.902 194.319 181.939 143.535 138.396 142.639 193.02 134.614 166.1 170.883 143.464 182.384 143.972 186.442 177.332 144.475 168.521 175.161 209.933 222.975 142.766 188.813 187.755 277.953 148.341 192.82 197.194 211.649 208.237 248.06 171.163 226.991 120.376 146.003 187.921 204.843 180.731 110.429

GGT 0.391 0.446 0.326 0.977 0.137 1.002 0.4 0.21 1.656 1.37 1.29 1.296 0.846 1.074 1.026 1.276 0.597 0.63 1.496 0.424 0.834 1.242 0.643 1.146 0.912 0.426 0.234 1.056 1.893 1.326 1.208 0.813 0.684 0.545 1.662 0.545 1.443 0.343 1.888 0.995 1.164 1.725 4.132 2.131 1.136 0.285 1.186 0.426 0.856 1.632 0.686 2.337 1.228 0.859 0.141 1.94 0.883 0.896

ALP 104.773 81.64 110.918 84.666 92.862 78.957 74.072 93.362 179.406 141.764 100.182 216.575 155.207 116.576 132.475 173.637 116.547 142.259 106.562 102.035 149.641 168.595 85.98 109.699 110.694 95.7 107.278 117.209 109.737 147.635 184.749 149.735 160.629 201.838 178.611 183.315 178.096 190.204 210.343 188.21 180.7 230.908 202.803 144.475 215.315 174.953 215.617 146.101 297.458 258.412 215.523 182.096 159.445 212.435 172.887 285.314 196.09 198.762

TC 1.548 1.412 1.334 1.699 1.422 1.209 1.645 1.307 2.926 3.224 3.409 4.257 4.303 3.09 2.618 3.048 3.661 2.834 3.25 2.409 3.222 2.606 2.256 3.666 2.884 2.454 2.757 1.631 3.538 3.138 2.979 2.86 1.957 3.509 2.845 2.975 3.671 2.272 3.677 2.456 3.507 4.217 3.235 3.035 2.775 4.93 3.117 3.341 2.236 4.19 2.313 2.034 1.924 2.785 4.273 3.542 2.595 3.871

TG 0.47 0.387 0.445 0.843 0.597 0.44 0.547 0.488 1.311 1.53 0.969 1.471 1.171 1.578 0.722 1.655 1.375 1.486 1.038 0.909 1.199 0.944 0.976 1.274 1.316 1.447 1.366 0.89 1.65 0.96 0.662 1.535 1.175 0.953 1.366 1.496 0.862 1.693 0.703 1.653 1.33 1.849 1.367 1.531 1.2 1.647 0.914 0.861 1.297 1.11 1.76 1.079 0.876 1.737 0.628 0.901 1.637 1.452

HDL-c 0.393 0.387 0.48 0.577 0.348 0.359 0.362 0.556 0.233 0.189 0.187 0.215 0.196 0.235 0.196 0.236 0.202 0.215 0.464 0.524 0.232 0.389 0.334 0.242 0.63 0.364 0.246 0.463 0.435 0.447 0.365 0.362 0.553 0.485 0.639 0.431 0.479 0.438 0.361 0.237 0.389 0.18 0.237 0.189 0.205 0.122 0.127 0.171 0.424 0.108 0.29 0.165 0.297 0.214 0.168 0.121 0.375 0.24

LDL-c 0.214 0.209 0.261 0.299 0.241 0.204 0.247 0.202 1.372 0.717 0.88 0.682 0.584 0.62 1.222 0.955 1.506 0.503 0.443 0.487 0.985 0.412 0.485 0.542 1.309 1.289 0.471 1.363 0.91 0.793 0.781 0.694 1.023 0.97 1.229 0.799 0.913 0.846 0.661 0.937 0.589 0.948 0.837 1.389 1.105 0.922 1.257 0.571 1.244 0.408 0.949 1.365 0.997 0.514 0.568 0.721 0.975 0.84

FBG 4.9 6 4.1 6.4 5.7 7.4 6.6 5.3 7.3 6.8 5.9 5.2 6.3 5.5 6.7 5.7 6.1 6 6.6 6 5.9 5.8 5 5.6 6.8 5.3 5.9 4.7 6.7 7.1 5.4 6.6 7 7.7 6.4 7.3 5.6 7.1 8.1 8 8 7.7 6.3 12.4 7.8 7 7.7 7.4 7.6 8.1 7.5 8.9 8.3 6.4 6.3 6.2 8 7.6

FINS 45.77011 61.04055 31.18005 47.92238 47.25221 50.2143 30.66333 14.10001 84.87921 72.95157 56.56021 65.11529 51.34835 76.17039 66.61576 30.77011 40.15395 41.47001 30.86981 49.29429 32.63586 29.43009 56.22119 43.86506 53.34894 44.93152 33.36204 57.94804 60.74108 54.6434 39.02504 59.89374 56.41859 40.44143 37.94328 74.10548 65.2749 63.54852 69.01889 73.72023 48.3786 54.40785 29.43009 81.54068 83.5338 56.53747 54.52559 88.51518 60.74108 43.30847 28.81712 33.89423 77.46974 67.38903 46.56021 64.7803 69.52268 37.329

HOMA-IR 9.967712 16.27748 5.681698 13.63126 11.97056 16.51492 8.994576 3.321336 27.53859 22.04759 14.83134 15.04887 14.37754 18.61943 19.83669 7.795093 10.88618 11.05867 9.055145 13.14514 8.557847 7.586424 12.4936 10.91753 16.12324 10.58387 8.74827 12.1047 18.08734 17.24303 9.36601 17.56883 17.55245 13.83996 10.79276 24.04311 16.2462 20.05309 24.8468 26.21164 17.20128 18.61958 8.240426 44.93798 28.95839 17.58944 18.65987 29.11166 20.51699 15.59105 9.605707 13.40705 28.57773 19.16843 13.03686 17.85057 24.71918 12.60891

TNF-α 3.47 4.71 2.89 3.47 3.79 2.31 3.47 4.21 6.95 6.37 8.63 10.01 9.47 10.47 7.53 8.63 9.21 7.63 5.21 4.47 5.79 8.69 7.53 8.89 6.01 3.29 3.75 6.29 6.05 12.31 7.53 10.73 7.00 6.47 7.63 8.10 7.89 9.63 12.31194 14.04686 12.31194 11.73436 8.462344 21.04583 12.88988 9.219542 8.625888 5.625768 5.205281 4.625888 6.365156 12.31194 10.15715 8.311942 7.157145 4.046857 4.625888 7.526482

IL-6 14.48 9.11 7.36 11.36 6.62 12.86 13.06 12.11 10.36 17.88 16.62 12.86 17.86 11.61 14.11 9.86 38.16 17.88 7.86 11.61 10.36 5.37 10.36 19.13 16.62 12.86 9.11 15.36 15.37 10.36 17.91 25.44 19.44 12.86 16.32 18.27 12.29 11.58 14.13104 27.7021 35.37387 15.36475 13.75274 20.94348 17.87587 10.35859 44.71275 48.87539 11.60812 39.43678 14.1112 11.60812 16.44302 27.97394 10.35859 34.78228 4.13104 10.35859

CRP 100.68 139.79 98.46 114.21 216.40 110.42 150.95 134.28 214.65 403.46 221.67 196.52 274.28 255.79 164.74 186.34 324.09 249.40 175.46 194.81 315.29 267.15 331.04 163.92 280.23 230.96 195.64 201.54 249.40 187.18 221.67 343.91 212.91 172.97 200.80 206.83 210.30 200.80 162.2839 220.7856 299.8331 167.1971 215.5246 361.2463 217.2746 180.4609 202.517 232.298 194.7645 221.6656 205.1019 113.4535 180.2273 219.9064 122.6285 205.9654 260.6998 286.5012

Liver TC 4.321957 13.21496 5.885217 4.23 4.873696 4.046087 6.988696 3.126522 13.86217 17.35652 26.71283 14.23 7.080652 22.39087 9.103696 13.21848 14.59783 13.31043 12.62087 6.804783 8.092174 6.988696 8.18413 7.356522 12.78196 3.770217 15.05761 6.213317 13.65916 8.103914 5.609348 5.241522 16.65477 3.770217 12.10446 10.54976 8.919783 6.107757 9.66136 13.43706 9.10611 13.43706 13.77021 9.379565 12.21551 14.43652 17.76802 6.885107 12.32656 18.32327 6.770217 22.98463 11.21606 9.931304 12.29891 17.98463 5.701304 8.092174

Liver TG 5.086881 18.08239 3.740354 3.815161 2.842669 3.740354 10.24857 6.957058 26.80744 21.14625 36.80744 19.20127 18.15397 23.31566 18.97685 17.55551 17.10667 12.18148 16.37839 8.827235 18.29312 12.11875 15.63468 7.92955 18.60281 12.11875 23.93826 7.031865 24.74034 24.91502 15.13418 16.19093 6.732637 26.65783 19.97385 22.00356 22.51457 19.11546 20.66132 36.92137 19.98693 45.66132 16.49356 40.84703 27.40146 32.07922 29.36859 30.51654 19.66785 42.97405 33.61854 33.3159 20.35215 20.32338 19.76014 31.39829 40.33109 26.13961

Liver Weight 11.3 10.4 12.2 11.8 13.3 10.8 11.6 10.3 19 19.2 22.1 25.3 25.2 18.8 18.6 19.2 21.6 23.9 11.9 19.1 19.4 21.5 16 18.8 16 16 15.5 17.1 14.8 15.7 24.4 26 18.8 22 20.9 21.4 27.8 24.1 18.3 18.5 24.6 19.6 24.9 25.1 28 24.1 28.3 32 21.9 23.3 21.2 19.1 23.5 24.3 20.3 27.3 25.2 26

Body Weight 451 433 455 462 510 480 449 433 569 479 600 588 666 582 559 564 554 620 478 519 522 568 604 517 494 561 602 612 468 462 581 559 505 561 550 572 627 652 524 545 601 533 520 562 680 589 597 634 495 523 556 558 591 547 547 609 565 682

Inflammation 0 0 0 0 0 0 0 0 1 1 1.666667 2.333333 1.333333 2 1.333333 1 2 2 0.666667 0 1.333333 1 1.666667 0.666667 0.333333 1 1 1.333333 1.666667 1 1.666667 1 1 2.333333 1 1 2 1 2.333333 2.666667 2.666667 2.333333 2.666667 2.333333 3 2 2.666667 3 2.333333 2.666667 1 2.666667 2 2.666667 2.666667 2 2 3

Steatosis 0 0 0 0 0 0 0 0 1.666667 2.333333 2.333333 1.333333 2.666667 1.333333 2.333333 3 2.666667 3 1 1.666667 0.333333 1 1 1 1.666667 2 1 2 1 2 2 1.333333 2.333333 1.666667 1 2 2 2 2.666667 3 3 2.333333 2.666667 3 3 3 3 3 2.333333 3 2.666667 2.666667 3 3 2.333333 3 3 3

Fiber Collagen Area 0 0 0 0 0 0 0 0 0.077619 0.033554 0.064229 0.06806 0.018612 0.039516 0.044048 0.051363 0.023835 0.04676 0.017864 0.028948 0.016597 0.030876 0.034356 0.025775 0.029277 0.027342 0.026627 0.026407 0.021666 0.012598 0.019974 0.026567 0.009431 0.058257 0.080591 0.031182 0.012857 0.030347 0.054173 0.078807 0.043844 0.062819 0.069726 0.096833 0.054436 0.053491 0.13017 0.071589 0.033764 0.06507 0.036292 0.080459 0.06758 0.063742 0.112451 0.153449 0.120056 0.081429

**Phylum**

Phylum NFD-1 NFD-2 NFD-3 NFD-4 NFD-5 NFD-6 NFD-7 NFD-8 HFD-1 HFD-2 HFD-3 HFD-4 HFD-5 HFD-6 HFD-7 HFD-8 HFD-9 HFD-10 FS-1 FS-2 FS-3 FS-4 FS-5 FS-6 FS-7 FS-8 FS-9 FS-10 CK-1 CK-2 CK-3 CK-4 CK-5 CK-6 CK-7 CK-8 CK-9 CK-10 PG-1 PG-2 PG-3 PG-4 PG-5 PG-6 PG-7 PG-8 PG-9 PG-10 BF-1 BF-2 BF-3 BF-4 BF-5 BF-6 BF-7 BF-8 BF-9 BF-10

Firmicutes 57.6236 37.9997 43.0918 57.9768 54.3692 46.8211 57.0917 44.6684 52.7371 44.235 71.0164 83.4013 57.9505 51.3259 75.1787 42.3311 47.5998 66.6496 55.9259 48.2466 54.4311 44.9125 24.8714 51.3351 45.281 28.6273 37.681 55.8994 45.5164 51.9261 61.1754 59.4486 43.6296 51.8164 55.1972 47.2971 45.9299 68.843 66.1011 55.8387 76.8517 56.0741 74.6347 58.2603 47.4275 78.2117 41.3173 55.3233 71.8479 78.551 55.197 60.4359 56.1618 48.6 49.4874 57.8624 63.1243 72.8723

Bacteroidetes 38.4044 55.3231 53.9055 36.2783 40.8978 43.6496 37.7446 50.4072 17.3022 41.9312 6.7303 2.2741 22.5435 27.6364 4.4187 42.6346 23.3926 11.4327 22.0477 43.5533 28.328 36.2302 35.0845 34.1668 21.7543 32.8134 34.4102 12.1371 31.5947 25.3147 22.7832 6.3232 37.9355 14.5757 28.955 36.0139 32.8935 16.4107 4.465 14.2185 7.6348 24.1133 12.6936 27.1104 34.7452 11.7638 29.8862 20.1429 15.028 12.201 23.0217 28.4519 2.2011 33.5648 40.3779 23.7296 24.7435 6.0769

Proteobacteria 1.6099 1.2948 1.2579 3.4834 1.6583 6.8759 1.7993 1.8893 14.3779 11.6043 13.6614 7.1232 13.6517 16.3431 13.7735 12.8996 23.8863 8.2604 7.7288 2.5375 13.6888 10.5499 13.0547 4.5852 14.0547 9.4784 14.6873 7.0127 7.3247 11.4597 11.6932 17.8822 14.2252 5.2033 10.7122 13.3353 17.7938 6.8472 11.607 11.6242 4.2387 7.7494 8.36 12.9012 14.9998 7.3243 12.3444 21.8258 10.7477 4.8055 7.7552 8.3344 19.1149 7.798 8.8849 15.3841 7.2567 4.0062

Verrucomicrobia 0.0608 3.7826 0.1651 0.3623 0.6533 0.2591 0.4248 0.0354 7.46 0.7066 2.8628 0.6714 3.4584 2.8308 0.9477 0.5476 1.9188 8.1073 8.1148 3.7112 0.4525 2.5367 20.7212 8.3053 17.4925 28.2897 11.3411 22.7948 12.6035 7.6872 1.949 11.8138 2.3526 26.276 1.0265 0.4032 0.8744 2.768 16.103 15.1793 9.6165 10.4794 0.4751 0.1864 0.118 0.725 14.1406 0.0577 1.2246 2.6844 12.3167 1.9724 20.4139 8.2828 0.1063 0.9835 3.6161 12.6915

Actinobacteria 0.4025 0.3533 0.123 0.3303 0.4959 0.5406 0.1382 0.8054 1.0055 0.3444 2.5548 2.7028 1.1159 1.324 4.9528 0.4119 1.0931 4.7874 0.644 0.9675 0.3652 0.4096 0.5621 1.2354 0.7792 0.473 0.8188 1.9985 1.172 1.6368 0.7072 2.297 1.0906 0.8466 2.5742 1.234 0.7187 2.7971 1.1051 1.5833 1.3084 1.036 1.0774 0.2676 0.8631 1.1547 0.283 1.5963 0.5275 0.7694 0.873 0.233 1.4698 0.5002 0.5352 0.707 0.6988 2.5299

Epsilonbacteraeota 0.1696 0.1277 0.0738 0.192 0.1484 0.045 0.2221 0.1542 5.1321 0.2642 2.1923 2.8272 0.1128 0.0347 0.3668 0.311 1.2454 0.3314 0.0895 0.052 0.1844 1.7737 3.2004 0.0689 0.0658 0.0293 0.6547 0.028 0.1677 1.5833 0.2678 0.5688 0.4197 0.0713 0.1976 0.191 0.4856 0.5101 0.0717 0.1711 0.1411 0.1486 1.8626 1.0287 0.92 0.1661 1.0437 0.1863 0.0887 0.4235 0.161 0.2156 0.05 0.2039 0.3291 0.5285 0.2296 0.1497

Cyanobacteria 0.5253 0.1463 0.4675 0.1073 0.7326 0.6374 0.5312 0.3283 0.2766 0.7818 0.1417 0.1235 0.9108 0.3017 0.1424 0.7427 0.4622 0.1167 1.2909 0.0485 0.2993 0.4021 0.5163 0.2634 0.5286 0.1866 0.3069 0.0783 0.3123 0.0487 0.3431 0.0869 0.1505 0.0591 0.1746 0.1334 0.1944 0.2001 0.2634 0.4127 0.0464 0.091 0.1988 0.169 0.353 0.1692 0.1994 0.2746 0.3491 0.1134 0.1738 0.2137 0.3337 0.3953 0.1886 0.2469 0.1013 0.5606

Fusobacteria 0.0246 0.0381 0.002 0 0.002 0.0137 0.002 0.0018 0.0069 0.0129 0.0273 0.0009 0.0126 0.0084 0.0374 0.0085 0.0026 0.0037 4.0709 0.7273 1.9942 3.0901 1.8459 0.001 0.0091 0.0192 0.0311 0.0058 0.0044 0.0067 0.001 0.0148 0.001 0.0281 0.0009 0.0152 0.0028 0.0036 0.0019 0.0039 0.001 0.0061 0 0.0019 0.0011 0 0.0211 0.028 0.0089 0.0151 0.1086 0.0019 0.1009 0.0019 0.0194 0.3214 9.00E-04 9.00E-04

Patescibacteria 0.7909 0.4378 0.5854 0.996 0.5121 0.5553 0.7021 0.9079 0.0286 0.0069 0.0173 0 0.0029 0.0075 0.0021 0.0009 0.0026 0.014 0 0.0364 0.0018 0.0021 0.0797 0.001 0.0037 0.0009 0.0194 0.0029 0.0062 0.0029 0.0076 0.0049 0.004 0.0009 0.0064 0.0051 0.0387 0.0009 0.062 0 0.0161 0 0.1113 0 0.0474 0.081 0.0294 0.0656 0.001 0.0879 0.0148 0 0.018 0.0452 0.0018 0.0173 0 0.0292

Deferribacteres 0 0.0041 0.001 0.001 0.001 0.002 0.0471 0.0009 0.5936 0 0.4306 0.5905 0.0058 0.0009 0.0031 0 0.189 0.0177 0.001 0.0026 0.0018 0 0 0 0 0.0146 0.0019 0.001 0.0204 0.0915 0.0048 0.0456 0.0289 0.0009 0.0064 0.004 0.0009 0.0755 0.0571 0 0.0101 0.0162 0.0676 0.0029 0.0285 0 0 0.0079 0.002 0.2552 0.3091 0 0.003 0.0058 0 0.0071 0.0425 0

Other 0.3883 0.4924 0.3271 0.2723 0.5293 0.6005 1.2967 0.8009 1.0796 0.1129 0.3652 0.2852 0.2351 0.1864 0.1767 0.1121 0.2078 0.2792 0.0867 0.117 0.253 0.0932 0.0639 0.0379 0.031 0.0675 0.0476 0.0416 1.2777 0.2424 1.0677 1.5142 0.1624 1.1217 1.149 1.3678 1.0673 1.5438 0.1627 0.9683 0.1352 0.2859 0.5189 0.0716 0.4964 0.4042 0.7349 0.4916 0.1746 0.0936 0.0691 0.1412 0.1329 0.6021 0.0694 0.2122 0.1863 1.0828

**Genus**

Genus NFD-1 NFD-2 NFD-3 NFD-4 NFD-5 NFD-6 NFD-7 NFD-8 HFD-1 HFD-2 HFD-3 HFD-4 HFD-5 HFD-6 HFD-7 HFD-8 HFD-9 HFD-10 FS-1 FS-2 FS-3 FS-4 FS-5 FS-6 FS-7 FS-8 FS-9 FS-10 CK-1 CK-2 CK-3 CK-4 CK-5 CK-6 CK-7 CK-8 CK-9 CK-10 PG-1 PG-2 PG-3 PG-4 PG-5 PG-6 PG-7 PG-8 PG-9 PG-10 BF-1 BF-2 BF-3 BF-4 BF-5 BF-6 BF-7 BF-8 BF-9 BF-10

Akkermansia 10.1608 9.6826 6.064 8.9499 11.1533 7.9591 13.4248 9.2354 7.9581 1.2056 3.3628 1.1714 3.9574 3.3224 1.4477 1.0476 2.4188 8.6073 8.6148 7.2112 3.9347 8.0367 21.2212 18.7983 17.9916 22.7897 11.8246 23.2948 13.1035 8.1051 3.449 12.3128 2.8526 26.776 1.4687 2.9022 1.3744 4.268 9.7329 5.0233 4.7216 4.4655 2.4858 1.7146 7.2317 3.1196 8.5894 14.5529 3.4994 3.2043 2.938 2.3002 10.4585 1.6237 2.4043 2.9903 2.0585 15.4235

Lactobacillus 15.4207 13.2219 15.8194 21.8083 17.2076 12.9222 12.3196 13.2991 4.2503 0.881 2.2789 1.8462 1.4477 0.7193 4.2297 0.9448 1.2772 1.104 11.3657 12.6386 15.303 9.1477 9.4379 11.7291 8.8028 9.3151 14.9797 9.7289 9.4358 19.9957 3.6623 6.3906 3.4726 13.0244 10.1378 5.1287 7.6702 10.1825 1.0673 3.3843 1.887 3.9197 3.9579 5.9915 4.4862 1.3946 3.6711 1.6943 2.4677 1.8461 1.2531 4.1659 2.5106 5.9212 3.1045 3.4458 1.0126 2.4784

Prevotella_9 5.0492 17.2318 9.9452 3.4315 8.4677 7.6481 14.4393 7.0918 2.5672 8.7128 6.5245 5.5322 9.6351 2.5375 8.5187 7.6235 3.5851 5.6214 3.8231 3.665 4.0959 2.775 2.0738 3.179 2.7764 3.428 2.2849 2.453 3.5444 2.5344 2.5629 3.5474 3.803 1.6613 4.738 5.8325 2.1201 4.5 9.3826 10.3726 10.8629 10.7045 10.7107 10.9705 10.1424 9.7609 9.916 9.6149 7.8007 8.5246 7.871 10.6241 7.032 8.3822 9.3285 9.2812 7.8389 7.1753

Eubacterium_coprostanoligenes_group 6.3215 4.2411 5.4265 6.7391 3.0191 4.9159 4.0997 6.583 6.4872 4.4837 7.5768 9.6017 4.9685 5.1916 3.6183 5.8297 6.1562 8.6812 7.6466 9.7668 4.3448 5.4093 5.1539 4.6852 5.2241 4.1875 5.6358 4.9299 4.9148 5.7267 13.1072 3.6789 5.6791 6.9689 10.4411 4.2788 5.567 5.8896 14.963 12.1567 15.1907 3.9065 5.7072 9.277 1.5692 6.71 3.6217 0.991 3.6527 6.486 4.3668 7.5684 3.2168 3.6743 5.1556 5.724 4.6029 3.5925

Bacteroides 3.1126 2.7887 3.9016 2.4425 4.5329 3.0204 2.4661 8.5444 17.2216 7.7767 3.9633 1.0444 9.1197 5.3558 2.463 6.6609 9.4854 2.4577 5.0868 5.6339 12.3033 6.1088 7.5237 3.3549 4.5716 6.3466 5.0516 2.7924 9.9584 1.381 9.2206 3.3066 18.2601 6.8423 2.0072 13.2319 8.8063 3.2098 4.103 8.1735 3.6165 6.4794 2.3727 2.1681 1.148 1.725 7.1584 4.9245 4.2246 3.6844 12.3147 1.9714 12.3959 8.2828 3.1063 4.9835 3.5908 12.6632

Bilophila 1.7445 1.671 1.6692 1.8499 1.7896 1.6251 1.7456 1.7494 7.1333 7.0701 8.2878 4.2123 6.8194 8.7615 7.2163 7.3527 10.3788 3.4884 2.5146 2.705 3.0874 3.4822 2.5116 1.9451 7.4922 4.6816 3.3907 3.8089 4.6068 1.0399 5.009 11.8605 4.8672 2.3572 1.8785 1.7532 3.9871 4.0673 7.1982 6.0809 9.5441 8.7063 6.8623 4.78 6.2092 8.7244 6.0682 5.9089 6.5527 8.85 9.0902 7.1662 9.9442 6.2579 7.4064 8.3021 6.6704 7.4417

Ruminiclostridium_9 2.7729 2.047 1.8878 2.4058 5.355 2.445 4.5986 2.3714 2.8221 3.7361 7.0183 6.4737 3.9393 5.3976 2.1995 3.8881 5.6032 6.6194 5.4426 4.6686 4.2733 5.6885 3.9093 7.87 4.854 5.5993 8.9351 5.4407 4.1937 5.3159 5.2396 3.59 3.1905 2.2682 9.9779 3.1438 3.2061 6.2423 5.1453 7.6639 5.3286 7.0417 7.2781 6.0539 8.4219 6.0819 8.3801 7.2672 4.5578 3.1352 3.3853 5.647 4.0839 5.2387 15.1087 10.7787 6.2098 2.4611

Alloprevotella 3.9991 2.6053 3.5863 3.9409 3.0038 5.2278 2.2553 2.0714 3.4962 13.7372 3.0826 2.6418 2.3147 12.3991 3.0082 11.9759 7.7533 6.7581 2.4742 3.692 2.4435 7.8084 2.3133 3.899 1.0378 2.2272 2.2809 1.9158 15.8369 3.7303 1.3292 2.4619 8.5666 3.0485 2.2866 12.573 8.154 4.5264 2.5783 3.7571 3.5392 3.5861 9.8042 6.0188 4.2724 6.5931 3.426 5.1545 5.8468 6.6533 4.0061 4.6402 3.2888 7.1178 3.1769 3.9566 3.8531 4.619

Lachnospiraceae_UCG_010 2.6603 2.5989 3.8578 2.6579 2.6108 2.6799 2.7456 2.7376 2.7648 1.5401 4.7083 4.9647 0.5388 3.2165 2.4182 3.4009 5.0791 5.0335 3.4947 4.1142 2.5628 1.9117 1.0871 3.4607 1.848 1.123 2.9312 3.3078 1.9152 4.0912 4.8463 7.8086 2.4077 2.9004 2.7983 2.004 5.2791 6.7817 4.8436 5.9225 5.7943 6.0013 6.0962 7.1577 9.5002 6.2603 4.814 4.907 5.258 3.3649 7.2131 5.9422 3.1439 5.3553 8.171 9.5415 9.0113 3.5852

Blautia 2.0019 1.6504 1.6907 1.9149 1.5864 1.6203 1.6771 2.0234 1.8506 2.9745 4.7734 6.3328 3.8452 3.4575 5.1756 2.8458 2.8873 2.978 2.5165 3.2035 2.3533 1.8778 1.7382 1.8842 1.7606 1.7177 1.6787 1.7736 2.4875 3.1437 2.4993 3.1906 2.8423 2.6725 2.8396 2.9165 2.5068 3.0243 4.2218 3.3039 3.2711 3.2483 4.4411 3.4586 3.2394 5.8632 3.0933 2.9849 2.7151 4.9169 2.3383 3.4564 2.3019 2.8841 2.4928 2.7289 3.4265 3.3447

Phascolarctobacterium 2.0081 2.5005 2.821 2.1917 2.3097 2.4377 2.026 4.627 1.2516 2.7187 1.3713 1.1801 3.3402 3.2502 1.6244 2.9137 2.0264 2.3065 5.5871 3.5368 5.5871 6.224 3.8695 3.5158 3.6428 4.1522 4.5009 4.6464 2.0375 0.6003 3.2372 1.1271 2.7419 1.6832 1.1047 2.8184 3.2835 1.433 2.2092 1.3532 1.0332 1.1553 2.0515 2.1347 1.8348 3.9431 0.6055 1.4721 1.3272 1.5946 1.425 1.575 0.1089 1.8593 1.1665 0.2234 2.6389 3.5534

Ruminococcaceae_NK4A214_group 2.2143 1.8883 2.1392 2.2402 1.8166 2.6152 1.7859 2.2762 1.4914 2.1061 3.1428 3.3411 3.8642 1.0078 2.204 2.4339 2.5614 2.8581 3.0145 2.7372 1.638 1.7482 1.6372 2.0934 2.5582 1.497 1.6316 2.0765 1.1342 1.5311 2.7895 3.6721 1.6664 1.4125 3.6592 2.1616 2.1423 2.4032 1.8635 1.4595 1.7872 2.1387 2.5753 3.488 1.0549 3.9196 1.8991 1.7546 2.4394 3.6278 2.1815 3.4314 0.5905 3.0174 1.7831 1.1529 2.9164 3.8473

Negativibacillus 1.1287 2.1576 1.285 1.0991 1.8158 1.1848 1.1453 1.2059 1.8413 1.7034 2.9058 2.3897 3.2012 2.3226 2.1045 1.7525 3.2079 1.9956 3.1741 2.7479 1.6205 1.9996 1.3402 2.5626 2.4352 1.5712 2.5359 3.1057 1.3677 2.6146 2.6282 5.3241 2.5087 2.3985 2.0237 2.0614 2.1355 4.4401 1.7066 1.5185 3.2781 1.9548 2.2383 1.8343 1.252 3.3658 0.7525 1.1222 2.2996 2.4065 3.2382 2.1039 3.0894 2.4653 2.0336 1.9272 1.2675 3.2347

Ruminococcaceae_UCG_014 3.7338 2.5983 4.5198 5.319 2.3229 5.5174 2.0987 5.6946 0.7183 1.2303 1.0606 2.0601 1.6858 2.1642 1.88427 1.3756 1.1419 1.9573 2.1373 2.7674 2.4882 2.5032 3.1329 2.4779 2.2417 2.7754 2.5286 1.9844 1.4598 2.3595 2.2617 2.1374 2.2587 1.6153 2.5184 1.855 1.7062 1.9922 4.7798 0.6167 0.1905 0.7116 0.7375 0.1304 2.5651 0.4338 0.645 8.4966 1.9977 0.4414 0.5036 0.3345 8.1881 0.1972 0.1192 1.565 1.6093 0.4017

Ruminococcaceae_UCG_005 1.5089 1.8777 2.018 1.2962 1.3445 1.6726 1.4585 3.7102 1.2329 2.9054 2.3065 2.6968 2.5927 3.0631 4.0729 3.0882 1.7252 1.4887 2.1473 2.0267 1.9568 1.6143 1.6233 2.4322 1.5975 1.572 2.759 2.45 1.5482 1.0469 3.9547 1.6174 2.7814 1.7538 2.4342 3.5136 3.5296 1.8839 1.5298 1.8706 1.4556 1.9152 2.211 1.3118 1.799 2.7628 1.677 2.5252 3.051 2.7621 1.4082 3.452 2.7673 1.542 1.4532 1.9404 2.6885 1.7983

Intestinimonas 1.3694 0.7883 0.7296 1.1247 2.3037 1.0978 2.0011 1.4755 1.2516 1.3204 2.2653 1.4096 2.0727 1.3218 1.3864 1.4104 1.8902 2.3289 1.5338 0.9699 1.453 1.8451 1.0362 1.0309 0.6985 0.6244 1.4218 0.9399 0.8944 1.2517 1.7353 0.9711 1.6404 1.2572 2.4644 1.6986 1.9078 0.9455 3.2954 3.4079 3.5786 4.4767 3.2415 3.2357 3.1064 3.3312 3.5485 3.5134 2.7855 2.9465 3.0435 2.7666 3.0336 2.7745 2.8531 2.8885 2.9535 2.956

Christensenellaceae_R-7_group 0.9785 0.8029 0.9644 1.0388 0.8323 1.0094 0.7733 1.8805 1.1943 3.5035 1.5448 2.0749 4.8691 2.3844 3.1323 3.9919 1.6944 3.6695 1.1286 2.1922 3.5844 1.209 0.5538 0.8922 0.6043 0.5768 0.9604 0.849 1.8104 1.6648 2.6987 2.4632 2.4718 2.751 3.9326 3.0124 1.9291 0.8819 0.5831 0.7578 0.628 0.9471 2.8705 2.4593 0.9706 1.8326 1.1108 1.5272 1.3738 1.5599 0.7367 1.5736 0.2319 1.6904 1.1694 1.4179 1.3506 0.2586

Collinsella 0.8391 0.953 0.9421 0.8154 0.6833 0.9462 1.0941 0.7726 1.4373 0.7771 2.7877 2.9915 1.4477 1.6282 4.8698 0.8025 1.4347 5.1044 0.939 1.3114 0.761 0.7318 0.7153 1.4869 1.0451 0.8376 1.1459 2.2964 1.5291 1.8014 1.1357 1.531 1.4779 1.2622 2.8068 1.4631 1.1348 2.9406 1.7613 2.3407 2.1421 1.845 1.9164 1.1874 1.6766 1.9209 1.1994 2.3444 1.3145 1.5407 1.2498 1.1769 1.8693 1.8011 1.9308 1.4673 1.4728 2.193

Megamonas 0.1521 0.269 0.2348 0.1724 0.2038 0.2974 0.2069 0.1983 0.127 0.1681 0.1163 0.1993 0.1092 0.1175 0.1649 0.1318 0.1791 0.1204 1.1751 1.1487 0.8309 2.362 1.018 3.3199 4.4189 6.014 1.5877 1.0264 0.6248 0.5821 0.9791 0.6624 0.593 1.0145 1.3379 2.0722 1.1427 2.4073 1.0726 1.2581 1.1351 1.6833 1.326 1.6849 2.8769 1.3518 1.4392 1.6857 1.283 1.3913 1.9273 2.3633 1.013 2.7535 2.4744 1.7386 2.5813 1.1315

Parabacteroides 2.0955 0.7081 1.0351 0.8819 1.5252 1.6546 1.3342 1.7454 2.3948 2.2897 1.699 1.6331 2.7247 2.1522 1.6008 2.2483 1.9001 1.7885 0.6223 0.7297 0.801 0.6143 0.9116 0.8123 1.2252 0.8659 0.8574 0.618 1.9381 0.5411 1.76 0.6511 1.8537 0.9303 0.6002 2.207 1.7181 1.0647 1.0542 0.6137 0.8428 1.1335 0.4924 0.6595 0.2115 1.048 1.6736 0.3422 0.4183 0.6342 0.1073 0.232 0.3048 0.1974 0.0915 0.5071 0.4152 0.1832

Kosakonia 0.5386 0.5567 0.6999 0.9965 0.5406 3.1964 0.5461 0.6252 3.5011 1.6079 2.563 2.3583 2.0968 1.6171 4.3297 1.7121 1.8397 1.8128 1.2104 0.7991 3.5755 0.5603 0.6375 1.3203 1.1905 0.6034 3.8452 0.6112 0.94 1.5024 0.5858 0.8101 0.8788 1.0203 1.6368 1.0174 3.1241 1.0292 0.0039 0.001 0 0 0.001 0.0309 0 0.0103 0.0028 0.0166 0.0059 0.0019 0.001 0.0077 0 0.1279 0.0749 0.002 0.1031 0

Prevotellaceae_UCG-001 2.0978 2.0746 4.7446 2.4523 4.4393 3.4003 4.1667 2.3048 0.0415 0.0534 0.0091 0.0017 0.0078 0.0216 0.0042 0.05 0.0094 0.014 0.0067 0.0078 0.0552 0.0434 0.4645 0.015 0.0091 0.0073 0.0282 0.0184 0.0381 0.0143 0.0257 0.0237 0.0768 0.0356 0.0184 0.0424 0.0212 0.0036 1.1622 2.0753 1.8417 3.2849 0.9999 1.8266 0.5301 3.0058 0.8462 1.9636 3.425 4.1108 1.0626 1.3285 0.5625 0.8676 0.428 0.3642 0.7015 0.9696

Alistipes 1.4873 1.2627 1.5501 1.3868 1.8066 1.7015 1.3633 1.6132 1.3166 0.4889 0.08 0.0626 0.4598 0.3214 0.0187 0.4392 0.1975 0.1111 0.2262 0.0737 0.1808 0.5228 1.811 0.1926 1.2657 3.8166 0.6372 0.4467 0.3221 0.0459 0.2735 0.0632 0.6579 0.3038 0.1305 0.4032 0.4294 0.2337 1.2664 1.0216 1.4491 0.8792 1.2687 1.0723 0.8577 1.2563 0.6318 1.1674 1.7147 1.0454 0.7861 0.5163 0.2058 0.5406 0.6128 1.5814 1.8462 1.5028

Desulfovibrio 0.7749 0.7132 0.5666 0.7126 0.7083 0.852 1.0998 0.6669 0.8921 1.1977 1.1242 0.567 1.8987 1.3537 0.8253 1.3435 1.2389 1.0872 0.8795 0.6445 0.876 0.7307 0.699 0.6053 0.5908 0.5215 1.0925 1.3144 1.3455 0.586 1.1529 1.452 1.0702 1.1778 0.8207 1.1711 0.9819 0.7673 0.3266 0.7394 0.3791 0.5948 0.4756 0.7538 0.7111 0.5635 0.7472 0.4592 0.5058 0.6995 0.5432 0.8721 0.031 0.7724 0.7358 0.3255 0.4846 1.0152

UBA1819 0.1123 0.0381 0.0543 0.0764 0.064 0.0841 0.2262 0.0789 0.321 0.2553 1.5899 2.0993 0.661 0.9867 2.1376 0.2601 0.4308 1.3593 0.4178 0.4257 0.1381 0.2392 0.0967 0.3562 0.4838 0.1418 0.2205 0.4805 0.4973 0.8062 0.674 0.6108 1.1334 0.3916 0.8679 1.119 0.6394 1.8497 1.7351 0.6967 1.9578 1.45 0.7441 0.6618 0.7307 0.8656 0.7433 1.0671 0.6889 0.7762 0.796 0.4628 0.4809 0.9175 0.6579 1.3968 1.1386 1.6745

Helicobacter 0.9661 0.1267 0.0738 0.1909 0.1473 0.045 0.2221 0.1542 5.1321 0.2632 2.1923 2.8167 0.1118 0.0347 0.3668 0.3101 1.2454 0.324 0.0866 0.052 0.1844 1.7737 3.2004 1.0689 0.0649 0.0293 0.6527 0.028 0.1668 1.5833 0.2659 0.5678 0.4197 0.0703 0.181 0.191 0.4846 0.5101 0.061 0.1673 0.1603 0.8958 0.2395 0.3574 0.8543 0.1374 0.8085 0.2703 1.2011 0.1153 0.5145 0.6942 0.019 1.0821 1.0815 0.6009 0.3878 0.0466

Clostridium_sensu_stricto_1 1.0211 0.0062 0.0123 0.031 0.0539 0.0176 0.0154 0.0472 1.2257 0.6495 1.1482 0.7122 0.4087 0.7137 0.8583 0.5141 0.9043 1.0075 0.0298 0.13 0.0294 0.0455 0.0159 0.1479 0.1284 0.054 0.0563 0.0909 0.1233 0.8342 0.6662 1.7815 0.3409 0.2522 0.375 1.2097 0.5657 1.2485 0.4517 0.6511 0.5211 0.5225 2.2426 1.4087 1.2979 0.5441 1.4237 0.4654 0.6887 0.3724 0.16 0.2156 0.05 0.2039 0.3291 0.5285 0.2296 0.1479

Oscillibacter 0.9793 0.172 0.1415 0.3313 0.8881 0.3031 1.1832 0.781 0.1531 0.1573 0.3007 0.1618 0.0885 0.1021 0.1839 0.1612 0.0885 0.2773 0.2558 0.2751 0.2543 0.4937 0.5199 0.3627 0.6216 0.9703 0.3321 0.4611 0.228 0.1529 0.2621 0.0484 0.4187 0.2203 0.3281 0.1759 0.1539 0.4156 0.6162 0.6189 0.7147 0.5869 0.661 0.6294 0.6444 0.7543 0.5413 0.5761 0.1795 0.3431 0.1699 0.614 4.9458 0.1741 0.5176 0.1765 0.1031 1.4489

Ruminococcus_torques_group 1.1643 0.0515 0.1046 0.0671 0.0406 0.1183 0.0819 0.1887 0.2617 0.2573 1.1547 2.7898 0.6182 0.3664 5.3841 0.2422 0.4418 0.2801 0.3494 0.7013 0.3393 0.3069 0.4057 0.2525 0.3887 0.2132 0.2467 0.3423 0.354 3.2268 0.1334 0.3565 0.2143 0.2916 0.2307 0.1354 0.2359 0.2437 0.0688 0.0826 0.0926 0.2449 0.0747 0.1656 0.1613 0.0445 0.1281 0.4647 0.0444 0.0879 0.0435 0.1518 0.017 0.2145 0.1581 0.1102 0.1338 0.0612

Romboutsia 0.2024 0.068 0.3619 0.3385 0.2682 0.3539 0.1464 0.1814 0.2677 0.6512 0.2589 0.5044 2.1141 0.4395 0.8511 0.5947 0.544 0.3352 0.2178 0.0904 0.1796 0.0904 0.0686 0.0962 0.174 0.0992 0.2562 0.0922 0.2626 0.1835 0.0762 0.4552 0.1894 0.1613 0.3832 0.2183 0.0875 0.7402 0 0.0435 0 0 0.0268 0.1265 0.019 0.0256 0.0239 0.0612 0.0118 0 0.001 0.0348 0 0.0029 0.0166 0 0.0362 0

other 21.384 22.6479 21.1529 21.0965 17.1591 21.4309 17.2532 14.0654 17.3955 23.5778 18.8022 24.1288 19.0413 24.2723 21.70083 22.6541 20.674 18.4246 21.8813 19.3337 18.7645 22.0958 19.2728 14.1528 18.256 11.6412 13.71 16.6758 11.3811 22.0071 21.7788 15.526 18.6643 11.477 23.5719 17.6887 23.9757 19.8741 12.2166 13.8672 14.0564 16.5205 14.8895 18.2503 21.6251 13.65 21.8144 11.7047 28.6687 23.8768 25.3245 24.611 15.1147 25.0604 21.8579 19.3531 30.6667 12.791

**SCFA**

Index NFD-1 NFD-2 NFD-3 NFD-4 NFD-5 NFD-6 NFD-7 NFD-8 HFD-1 HFD-2 HFD-3 HFD-4 HFD-5 HFD-6 HFD-7 HFD-8 HFD-9 HFD-10 FS-1 FS-2 FS-3 FS-4 FS-5 FS-6 FS-7 FS-8 FS-9 FS-10 CK-1 CK-2 CK-3 CK-4 CK-5 CK-6 CK-8 CK-9 CK-10 PG-1 PG-2 PG-3 PG-4 PG-5 PG-6 PG-7 PG-8 PG-9 PG-10 BF-1 BF-2 BF-3 BF-4 BF-5 BF-6 BF-7 BF-8 BF-9 BF-10

AA 0.666 1.052 0.576 0.668 0.304 0.428 0.946 0.218 0.01278 0.1192 0.099 0.1078 0.1138 0.0458 0.1404 0.065 0.0588 0.926 0.206 0.452 0.496 0.268 0.24 0.28 0.1526 0.18 0.21 0.76 0.24 0.582 0.61 0.636 0.506 0.334 0.26 0.1132 0.222 0.18 0.236 0.174 0.0508 0.186 0.296 0.13 0.0474 0.103 0.225 0.0864 0.106 0.137 0.0704 0.0705 0.365 0.0665 0.251 0.193 0.0915

IBA 0.0596 0.0562 0.0326 0.0368 0.0388 0.0462 0.0456 0.0258 0.01562 0.023 0.027 0.0284 0.0248 0.01418 0.0252 0.01588 0.0186 0.079 0.0298 0.0636 0.0626 0.0328 0.0298 0.0462 0.0516 0.0384 0.0354 0.0746 0.0548 0.0546 0.103 0.082 0.0792 0.0452 0.0342 0.0214 0.0386 0.0168 0.0336 0.0271 0.0171 0.0268 0.0276 0.0258 0.0118 0.0225 0.0243 0.0181 0.0161 0.0117 0.0136 0.0181 0.0328 0.014 0.0223 0.0141 0.0167

BA 0.0516 0.63 0.246 0.056 0.0508 0.1472 0.0476 0.038 0.0434 0.0764 0.0606 0.1064 0.075 0.0464 0.0954 0.065 0.0552 0.1774 0.1016 0.238 0.246 0.096 0.0488 0.153 0.0676 0.098 0.1084 0.1976 0.1154 0.1696 0.258 0.218 0.1526 0.183 0.095 0.1028 0.115 0.0534 0.169 0.0773 0.0495 0.115 0.063 0.0725 0.0385 0.0699 0.0888 0.065 0.0328 0.0534 0.0541 0.048 0.0823 0.0492 0.0816 0.0575 0.0635

IVA 0.0286 0.0834 0.0278 0.0436 0.0706 0.184 0.0492 0.034 0.0148 0.0206 0.0212 0.03 0.0242 0.01476 0.0274 0.01738 0.01766 0.0436 0.0248 0.054 0.0578 0.0276 0.0306 0.0444 0.0308 0.0314 0.0354 0.0638 0.0488 0.051 0.0796 0.0558 0.0612 0.0366 0.0312 0.0226 0.0252 0.00971 0.0302 0.0211 0.016 0.0192 0.0145 0.0204 0.00962 0.0217 0.0149 0.0146 0.0108 0.0124 0.0143 0.0167 0.0162 0.0136 0.0148 0.00668 0.0162

VA 0.0248 0.0492 0.0394 0.053 0.0458 0.0348 0.0466 0.0294 0.01644 0.0328 0.0278 0.049 0.0358 0.01956 0.047 0.0256 0.0218 0.1016 0.0408 0.0732 0.0656 0.029 0.0218 0.0458 0.0246 0.0294 0.0454 0.0844 0.0538 0.085 0.0734 0.0954 0.0686 0.056 0.0378 0.0254 0.044 0.0244 0.0435 0.033 0.0148 0.0281 0.0246 0.0219 0.0122 0.0238 0.0217 0.0214 0.0178 0.0188 0.0199 0.0197 0.02 0.0208 0.0265 0.0183 0.0238

HA 0.0041 0.00348 0.0053 0.00434 0.00494 0.00378 0.00388 0.00542 0.00406 0.00418 0.00426 0.0043 0.00438 0.00368 0.00352 0.0042 0.00378 0.00558 0.0039 0.00468 0.0033 0.00542 0.00402 0.00562 0.00328 0.00542 0.00488 0.00412 0.0063 0.00392 0.0044 0.00374 0.00346 0.00422 0.00388 0.0043 0.0042 0.00266 0.0031 0.00284 0.002 0.00251 0.00161 0.00216 0.00181 0.00294 0.00193 0.00238 0.00208 0.00349 0.00323 0.00224 0.00198 0.00312 0.00309 0.00204 0.00337

PA 0.1802 0.142 0.494 0.1864 0.2272 0.1586 0.109 0.1708 0.00866 0.1848 0.105 0.0952 0.0486 0.01446 0.0894 0.0264 0.0386 0.288 0.1602 0.342 0.294 0.1464 0.0326 0.1878 0.129 0.0888 0.1278 0.366 0.206 0.248 0.396 0.498 0.244 0.224 0.26 0.0486 0.1528 0.125 0.169 0.158 0.0302 0.15 0.151 0.11 0.0165 0.0728 0.153 0.0779 0.0964 0.0323 0.0461 0.115 0.184 0.0292 0.173 0.13 0.0498

Total SCFAs 1.0149 2.01628 1.4211 1.04814 0.74214 1.00258 1.24788 0.52142 0.11576 0.46098 0.34486 0.4211 0.32658 0.15884 0.42832 0.21946 0.21444 1.62118 0.5671 1.22748 1.2253 0.60522 0.40762 0.76282 0.45948 0.47142 0.56728 1.55052 0.7251 1.19412 1.5244 1.58894 1.11506 0.88302 0.72208 0.3383 0.6018 0.41197 0.6844 0.49334 0.1804 0.52761 0.57831 0.38276 0.13783 0.31664 0.52963 0.28578 0.28198 0.26909 0.22163 0.29024 0.70228 0.19642 0.57229 0.42162 0.26487

**Bile acids**

Index NFD-1 NFD-2 NFD-3 NFD-4 NFD-5 NFD-6 NFD-7 NFD-8 HFD-1 HFD-2 HFD-3 HFD-4 HFD-5 HFD-6 HFD-7 HFD-8 HFD-9 HFD-10 FS-1 FS-2 FS-3 FS-4 FS-5 FS-6 FS-7 FS-8 FS-9 FS-10 CK-1 CK-2 CK-3 CK-4 CK-5 CK-6 CK-8 CK-9 CK-10 PG-1 PG-2 PG-3 PG-4 PG-5 PG-6 PG-7 PG-8 PG-9 PG-10 BF-1 BF-2 BF-3 BF-4 BF-5 BF-6 BF-7 BF-8 BF-9 BF-10

GCDCA 1.82E+02 1.24E+02 5.80E+01 1.12E+02 1.32E+02 6.54E+02 4.36E+02 2.62E+02 9.60E+02 9.78E+02 8.82E+02 3.18E+02 2.66E+03 8.58E+02 2.64E+02 4.14E+02 9.42E+02 9.96E+02 4.50E+02 2.62E+02 1.02E+02 9.44E+01 7.22E+01 1.32E+02 1.27E+03 4.96E+02 4.22E+02 1.04E+03 3.28E+02 2.54E+03 1.86E+02 1.21E+02 5.02E+02 8.92E+01 4.48E+02 2.78E+02 3.64E+02 5.82E+03 3.98E+02 6.96E+02 1.51E+03 8.04E+02 4.06E+02 4.36E+02 3.17E+02 3.43E+03 3.40E+02 235.2 592.0 1164.0 404.0 3536.0 536.0 424.0 149.6 560.0 268.0

TCA 5.96E+03 4.20E+03 4.34E+03 4.06E+03 1.66E+03 3.48E+03 5.06E+03 3.92E+03 3240 4460 3140 2000 2820 4660 2640 4340 4988 2160 3040 4360 1840 4340 4420 1662 3600 3020 3520 2480 2820 2560 3100 3540 9920 4020 3460 1776 2320 2280.0 2740.0 7906.0 6560.0 7300.0 10360.0 4280.0 7060.0 7546.0 4720.0 3080.0 5640.0 4340.0 6060.0 4916.0 6460.0 5390.0 2300.0 8260.0 10700.0

Tβ_MCA 1.16E+03 9.96E+02 1.15E+03 1.50E+03 1.28E+03 1.60E+03 1.95E+03 2.16E+03 7.16E+02 4.06E+03 1.52E+03 1.13E+03 1.16E+03 1.42E+03 1.11E+03 2.64E+02 4.92E+02 1.10E+03 2.36E+03 1.08E+03 1.02E+03 1.15E+03 1.36E+03 1.48E+03 1.60E+03 5.52E+02 2.16E+03 5.84E+03 4.10E+03 2.34E+03 8.70E+02 1.12E+03 1.20E+04 1.24E+03 2.78E+03 4.04E+02 1.39E+03 3300.0 3462.0 3372.0 1632.0 9420.0 4062.0 3234.0 1998.0 2280.0 5598.0 2676.0 1732.0 1152.0 1320.0 1544.0 2340.0 1444.0 1560.0 1332.0 4680.0

CA 1.65E+04 1.41E+04 4.54E+04 3.44E+04 6.26E+04 1.31E+04 2.59E+04 5.86E+04 2.40E+03 4.08E+03 5.62E+03 1.29E+03 1.13E+04 4.40E+03 1.17E+04 1.37E+03 1.85E+03 1.10E+04 1.15E+04 5.64E+03 4.06E+03 5.38E+03 7.84E+04 2.62E+03 2.48E+03 4.74E+03 5.86E+04 7.06E+04 2.44E+04 7.72E+04 1.38E+04 2.68E+03 7.28E+04 3.68E+03 7.76E+03 1.85E+03 3.12E+03 3.20E+03 4.24E+03 4.18E+03 1.85E+03 4.16E+04 2.36E+04 1.64E+03 1.79E+03 5.84E+03 5.60E+03 8.66E+04 3.72E+03 4.70E+03 1.56E+03 3.30E+03 3.14E+03 0.00E+00 2.00E+03 3.82E+03 3.28E+04

TCDCA 2.96E+02 1.06E+03 4.36E+02 2.94E+02 0.00E+00 7.18E+02 3.28E+02 1.64E+03 4.64E+02 2.72E+03 1.49E+03 5.62E+02 9.80E+02 1.21E+03 1.27E+03 3.76E+02 3.06E+02 9.08E+02 1.96E+03 2.96E+02 0.00E+00 5.56E+02 1.01E+03 2.34E+02 7.52E+02 3.28E+02 2.02E+03 2.46E+03 9.22E+02 3.10E+03 5.36E+02 3.54E+02 4.08E+03 4.34E+02 1.56E+03 3.36E+02 5.24E+02 7.10E+02 3.64E+02 4.92E+02 4.82E+02 9.22E+02 1.67E+03 4.66E+02 3.46E+02 5.14E+02 6.00E+02 1048.0 2220.0 1728.0 800.0 1308.0 1632.0 584.0 712.0 1064.0 836.0

GCA 2.49E+03 1.34E+03 6.46E+02 9.82E+02 1.73E+03 2.72E+03 1.16E+03 1.70E+03 3.88E+03 2.26E+03 4.24E+03 1.67E+03 3.92E+03 2.80E+03 7.58E+02 2.78E+03 1.19E+03 7.32E+03 1.66E+03 4.34E+02 5.42E+02 6.46E+02 1.78E+03 5.30E+02 3.02E+03 1.16E+03 1.30E+03 1.49E+03 1.21E+03 7.20E+03 1.55E+03 1.39E+03 6.98E+03 1.03E+03 8.24E+02 8.90E+02 1.05E+03 10260.0 1686.0 2184.0 7620.0 3246.0 3600.0 1350.0 2520.0 0.0 1872.0 2632.0 3968.0 4888.0 2856.0 0.0 4800.0 3024.0 1496.0 2808.0 2576.0

β_MCA 1.95E+06 1.57E+06 1.39E+06 2.20E+06 1.08E+06 1.89E+06 2.45E+06 2.06E+06 1.10E+06 6.74E+05 1.79E+06 1.16E+06 1.76E+06 1.36E+06 1.52E+06 1.40E+06 1.26E+06 1.02E+06 1.23E+06 1.25E+06 1.57E+06 1.39E+06 1.60E+06 1.26E+06 1.49E+06 1.56E+06 2.06E+06 1.83E+06 1.30E+06 9.34E+05 9.24E+05 1.00E+06 1.02E+06 1.68E+06 1.57E+06 8.34E+05 1.05E+06 650000 704000 708000 510000 923000 887000 760000 690000 593000 860000 968000 1120000 1202000 962000 1094000 1272000 1028000 716000 1186000 1428000

HCA 8.10E+04 3.37E+05 7.50E+04 2.52E+05 1.25E+05 1.96E+05 1.35E+05 1.10E+05 5.90E+04 1.90E+04 1.59E+05 6.46E+04 1.38E+05 8.24E+04 7.96E+04 7.88E+04 7.74E+04 2.58E+04 2.86E+05 8.10E+04 1.37E+05 7.50E+04 4.42E+05 8.48E+04 1.08E+05 1.17E+05 1.08E+05 1.26E+05 1.37E+05 6.76E+04 3.48E+04 9.66E+04 5.42E+04 9.66E+04 6.84E+04 2.90E+04 1.40E+05 6.88E+04 9.46E+04 1.26E+05 7.56E+04 1.32E+05 9.98E+04 5.78E+04 9.62E+04 1.08E+05 4.94E+04 8.94E+04 5.94E+04 4.48E+04 8.96E+04 6.62E+04 5.04E+04 1.40E+05 3.28E+04 8.74E+04 1.79E+05

α_MCA 5.88E+05 7.88E+05 6.40E+05 1.02E+06 8.68E+05 5.68E+05 7.74E+05 1.11E+06 4.06E+05 1.95E+05 8.78E+05 6.60E+05 1.01E+06 7.66E+05 6.38E+05 5.62E+05 6.62E+05 3.36E+05 9.16E+05 5.68E+05 7.88E+05 6.40E+05 9.00E+05 6.68E+05 5.68E+05 6.54E+05 9.52E+05 9.06E+05 5.44E+05 5.22E+05 3.70E+05 5.50E+05 5.40E+05 6.88E+05 7.34E+05 3.44E+05 6.74E+05 6.62E+05 9.82E+05 8.84E+05 5.44E+05 9.86E+05 9.66E+05 7.52E+05 7.44E+05 7.34E+05 6.44E+05 6.50E+05 5.82E+05 6.16E+05 6.16E+05 5.34E+05 7.16E+05 8.26E+05 3.98E+05 7.62E+05 8.22E+05

CDCA 0.00E+00 0.00E+00 0.00E+00 5.52E+04 5.78E+04 0.00E+00 0.00E+00 6.88E+04 0 0 0 0 35400 0 0 0 0 0 494000 0 0 355000 71200 0 0 0 48200 232000 73000 674000 0 0 292000 0 23000 0 0 0 0 0 0 0 0 0 0 0 0 0 0 0 0 0 0 0 0 0 0

GHCA 0.00E+00 0.00E+00 0.00E+00 0.00E+00 0.00E+00 0.00E+00 0.00E+00 0.00E+00 9.42E+02 0.00E+00 0.00E+00 0.00E+00 0.00E+00 0.00E+00 0.00E+00 0.00E+00 0.00E+00 0.00E+00 0.00E+00 0.00E+00 0.00E+00 0.00E+00 0.00E+00 0.00E+00 0.00E+00 0.00E+00 0.00E+00 0.00E+00 1.08E+03 0.00E+00 0.00E+00 0.00E+00 0.00E+00 0.00E+00 0.00E+00 0.00E+00 0.00E+00 0.0 0.0 0.0 0.0 0.0 0.0 0.0 0.0 0.0 0.0 0.0 0.0 0.0 0.0 0.0 0.0 0.0 0.0 0.0 0.0

12_KLCA 1.24E+05 5.12E+04 6.24E+04 9.78E+04 1.19E+05 7.30E+04 5.78E+04 7.76E+04 2.32E+04 3.22E+04 5.24E+04 2.58E+04 8.12E+04 3.36E+04 3.32E+04 3.68E+04 1.96E+04 2.10E+04 1.01E+05 4.52E+04 5.92E+04 6.78E+04 1.38E+05 3.88E+04 7.46E+04 5.20E+04 7.76E+04 7.00E+04 5.14E+04 6.46E+04 2.10E+04 3.94E+04 5.28E+04 5.34E+04 7.00E+04 2.24E+04 5.10E+04 3.74E+04 5.22E+04 4.02E+04 3.30E+04 3.96E+04 5.38E+04 3.84E+04 5.72E+04 4.16E+04 3.22E+04 2.68E+04 5.70E+04 5.60E+04 3.12E+04 2.24E+04 2.62E+04 6.00E+04 1.84E+04 2.92E+04 5.36E+04

DCA 6.34E+04 8.46E+04 1.38E+05 1.18E+05 9.04E+04 1.09E+05 5.36E+04 1.03E+05 99400 70400 180200 91200 122200 126800 106600 119400 139600 106200 145400 123400 118600 137600 105600 126400 108800 137600 102800 111400 108400 112000 112200 75800 104800 100400 118000 83000 96800 132000 124400 105200 132000 102400 125200 90200 92800 114400 128000 75200 106200 94000 95400 101800 110800 88800 97000 128400 104800

GDCA 9.84E+02 4.82E+02 1.24E+03 2.12E+03 1.64E+03 2.70E+02 6.46E+02 4.68E+02 2.78E+03 2.36E+03 6.92E+03 4.38E+03 4.52E+03 1.68E+03 1.04E+03 2.44E+03 2.92E+03 7.56E+03 7.18E+02 8.86E+02 1.14E+03 1.24E+03 2.56E+03 1.60E+03 4.70E+02 6.46E+02 8.68E+02 1.17E+03 3.34E+03 8.28E+03 2.40E+03 1.97E+03 4.50E+03 2.48E+03 1.21E+03 8.58E+02 9.18E+02 6720.0 6320.0 1588.0 1992.0 3064.0 1016.0 2252.0 1800.0 6520.0 4760.0 832.0 1616.0 2308.0 3844.0 1036.0 1424.0 3544.0 1080.0 2116.0 4160.0

GLCA 2.16E+02 8.92E+02 8.56E+02 1.02E+03 9.52E+02 4.32E+02 2.52E+02 4.78E+02 4.08E+02 6.26E+02 3.64E+02 4.20E+02 2.02E+02 3.02E+02 1.75E+02 3.76E+02 4.64E+02 1.20E+03 3.62E+02 2.16E+02 1.02E+03 8.16E+02 1.02E+03 9.52E+02 5.54E+02 2.92E+02 4.78E+02 3.08E+02 8.56E+02 1.27E+03 4.36E+02 3.46E+02 2.64E+02 6.82E+02 5.94E+02 1.72E+02 4.34E+02 1.10E+03 9.34E+02 5.96E+02 2.58E+02 7.10E+02 4.14E+02 3.98E+02 2.48E+02 4.86E+02 9.96E+02 205.2 371.6 436.0 792.0 220.8 600.0 616.0 456.0 788.0 1112.0

GUDCA 5.22E+02 2.04E+02 1.34E+02 4.50E+02 3.16E+02 2.08E+02 1.14E+02 1.66E+02 3.08E+02 5.32E+02 3.14E+02 1.52E+02 2.36E+02 1.47E+02 4.08E+02 2.46E+02 2.40E+02 6.22E+02 2.44E+02 5.42E+02 2.44E+02 9.40E+01 5.30E+02 2.36E+02 2.28E+02 1.76E+02 2.18E+02 4.04E+02 5.42E+02 1.10E+03 3.14E+02 2.88E+02 3.48E+02 1.91E+02 3.36E+02 1.46E+02 2.20E+02 6.32E+02 2.34E+02 3.62E+02 2.82E+02 1.50E+02 6.40E+01 1.68E+02 7.40E+01 1.65E+03 4.64E+02 158.8 696.0 1804.0 306.4 3188.0 234.0 318.0 258.8 262.8 330.0

LCA 1.76E+05 2.14E+05 1.75E+05 5.70E+04 1.10E+05 7.38E+04 2.44E+05 1.12E+05 1.04E+05 1.55E+05 1.77E+05 1.71E+05 1.94E+05 1.55E+05 2.28E+05 2.16E+05 1.76E+05 2.08E+05 1.85E+05 2.12E+05 2.14E+05 1.75E+05 9.70E+04 1.50E+05 1.54E+05 3.18E+05 1.42E+05 2.80E+05 8.28E+04 1.18E+05 1.19E+05 8.14E+04 7.14E+04 1.22E+05 2.60E+05 1.18E+05 5.82E+05 1.06E+05 1.49E+05 9.11E+04 1.84E+05 8.18E+04 8.62E+04 7.55E+04 9.62E+04 1.52E+05 3.89E+05 5.42E+04 1.64E+05 1.33E+05 1.59E+05 9.08E+04 1.25E+05 8.80E+04 1.55E+05 1.16E+05 1.49E+05

TDCA 1.72E+03 9.20E+02 1.38E+03 2.26E+03 1.15E+03 1.75E+03 5.04E+02 4.52E+02 1.13E+04 3.14E+03 3.70E+03 3.80E+03 2.52E+03 1.52E+03 2.36E+03 2.04E+03 1.72E+04 1.75E+04 5.62E+02 2.52E+03 1.32E+03 1.38E+03 3.86E+03 1.15E+03 1.55E+03 5.04E+02 6.78E+02 7.54E+02 4.86E+03 4.12E+03 8.18E+02 1.11E+03 7.98E+02 1.34E+03 3.92E+03 4.56E+02 8.90E+02 3.94E+03 2.64E+03 2.12E+03 6.24E+02 4.02E+03 1.58E+03 7.52E+02 9.20E+02 1.33E+03 3.20E+03 1368.0 4720.0 5344.0 2688.0 3488.0 10240.0 2480.0 2120.0 3712.0 12240.0

TUDCA 6.46E+02 2.30E+02 1.24E+02 2.78E+02 1.70E+02 2.52E+02 9.50E+01 9.92E+01 1.09E+03 3.36E+02 2.56E+02 5.84E+02 5.48E+02 2.36E+02 3.90E+02 2.28E+02 9.44E+02 2.48E+03 1.45E+02 1.45E+03 2.30E+02 1.23E+02 2.98E+02 1.70E+02 5.28E+02 9.50E+01 9.92E+01 3.72E+02 9.18E+02 2.04E+02 3.06E+02 3.14E+02 1.01E+03 3.00E+02 8.70E+02 1.09E+02 2.70E+02 3.04E+02 3.60E+02 2.98E+02 1.82E+02 5.26E+02 2.94E+02 2.04E+02 1.88E+02 5.32E+02 7.28E+02 370.0 716.0 632.0 359.6 984.0 548.0 532.0 400.0 436.0 592.0

UDCA 3.68E+05 4.42E+05 2.76E+05 2.56E+05 1.92E+05 2.58E+05 3.12E+05 2.98E+05 238000 216000 156000 184000 218000 96000 254000 124000 157200 272000 290000 238000 262000 288000 240000 354000 278000 300000 298000 276000 288000 342000 234000 210000 167800 300000 320000 185400 330000 186000 144000 222000 262000 288000 75800 186000 204000 230000 484000 126600 171000 276000 308000 118600 236000 142000 222000 234000 76400

ω_MCA 2.34E+06 1.56E+06 1.79E+06 1.89E+06 9.62E+05 6.84E+05 1.12E+06 1.25E+06 7.86E+05 8.46E+05 1.37E+06 8.76E+05 1.48E+06 1.17E+06 7.98E+05 1.24E+06 9.30E+05 8.98E+05 1.74E+06 1.34E+06 1.56E+06 1.39E+06 1.89E+06 8.02E+05 8.84E+05 1.12E+06 1.21E+06 9.30E+05 8.24E+05 6.76E+05 1.27E+06 1.27E+06 3.70E+05 1.03E+06 7.98E+05 7.18E+05 1.36E+06 1.19E+06 1.84E+06 1.41E+06 1.45E+06 1.37E+06 1.13E+06 1.22E+06 1.07E+06 1.43E+06 1.48E+06 1.22E+06 1.17E+06 1.34E+06 1.39E+06 1.10E+06 1.28E+06 1.51E+06 1.02E+06 1.31E+06 1.19E+06

TLCA 3.24E+02 1.92E+02 1.34E+02 2.56E+02 1.60E+02 1.71E+02 7.56E+01 9.22E+01 9.26E+02 4.74E+02 3.62E+02 4.26E+02 4.98E+02 3.24E+02 3.32E+02 2.18E+02 3.74E+02 5.08E+03 8.68E+01 7.24E+02 2.12E+02 2.14E+02 3.36E+02 1.60E+02 1.71E+02 7.56E+01 8.22E+01 2.62E+02 5.94E+02 3.54E+02 7.66E+02 2.68E+02 9.34E+02 2.22E+02 6.92E+02 9.18E+01 3.04E+02 5.92E+02 6.82E+02 2.70E+02 1.60E+02 3.64E+02 9.76E+01 3.76E+02 1.04E+02 4.34E+02 1.03E+03 212.8 248.0 272.0 548.0 515.2 484.0 678.4 328.8 400.0 1832.0

7_KLCA 2.96E+04 4.56E+04 3.12E+04 3.64E+04 3.78E+04 4.64E+04 3.84E+04 0.00E+00 1.94E+04 0.00E+00 0.00E+00 0.00E+00 0.00E+00 0.00E+00 0.00E+00 0.00E+00 2.16E+04 0.00E+00 0.00E+00 3.76E+04 5.16E+04 4.70E+04 0.00E+00 3.78E+04 6.64E+04 6.64E+04 0.00E+00 3.80E+04 6.92E+04 1.31E+05 7.56E+04 3.14E+04 5.92E+04 8.50E+04 6.60E+04 4.86E+04 4.00E+04 #VALUE! 4.36E+04 0 14533 0 21200 17933 30400 26467 0 0 31733 0 49000 0 29400 28800 9650 23600 0

ConBAs 1.45E+04 1.06E+04 1.05E+04 1.33E+04 9.19E+03 1.23E+04 1.06E+04 1.14E+04 27034 21946 23184 15449.8 20064 15159.2 10737.4 13722 30100 46926 11591.6 12770 7671.6 10653.4 17244.2 8308.8 13743.4 7342.4 11841.4 16582 21578 33066 11283.8 10820.6 41314 12034.4 16694 5517.2 8692 43538 25099.8 24124 22552 38566.2 26725.8 15420 17414.8 27384 30704.2 12818 22519.6 24068 19978 20736 29298 19034.4 10861.2 21738.8 39326

UnConBAs 5.74E+06 5.11E+06 4.61E+06 6.01E+06 3.70E+06 3.91E+06 5.21E+06 5.25E+06 2839380 2211440 4772220 3238088 5057700 3792200 3665100 3778368 3447092 2902020 5398520 3908840 4772060 4562580 5554000 3528620 3731680 4331540 5056800 4874000 3506600 3718600 3170780 3363280 2803000 4167280 4035160 2384254 4335120 3035400 4104973 3586680 3213650 3980333 3475800 3210003 3048190 3411040 4099933 3292800 3453120 3762100 3702164 3135100 3851140 3915600 2675050 3884020 4038800
